# High-speed 2D light-sheet fluorescence microscopy enables quantification of spatially varying calcium dynamics in ventricular cardiomyocytes

**DOI:** 10.3389/fphys.2023.1079727

**Published:** 2023-02-14

**Authors:** Liuba Dvinskikh, Hugh Sparks, Kenneth T. MacLeod, Chris Dunsby

**Affiliations:** ^1^ Department of Physics, Imperial College London, London, United Kingdom; ^2^ National Heart and Lung Institute, Imperial College London, London, United Kingdom; ^3^ Department of Chemistry, Imperial College London, London, United Kingdom

**Keywords:** ventricular cardiomyocyte, cardiac electrophysiology, calcium imaging, light-sheet fluorescence microscopy, live cell imaging

## Abstract

**Introduction:** Reduced synchrony of calcium release and t-tubule structure organization in individual cardiomyocytes has been linked to loss of contractile strength and arrhythmia. Compared to confocal scanning techniques widely used for imaging calcium dynamics in cardiac muscle cells, light-sheet fluorescence microscopy enables fast acquisition of a 2D plane in the sample with low phototoxicity.

**Methods:** A custom light-sheet fluorescence microscope was used to achieve dual-channel 2D timelapse imaging of calcium and the sarcolemma, enabling calcium sparks and transients in left and right ventricle cardiomyocytes to be correlated with the cell microstructure. Imaging electrically stimulated dual-labelled cardiomyocytes immobilized with para-nitroblebbistatin, a non-phototoxic, low fluorescence contraction uncoupler, with sub-micron resolution at 395 fps over a 38 μm × 170 µm FOV allowed characterization of calcium spark morphology and 2D mapping of the calcium transient time-to-half-maximum across the cell.

**Results:** Blinded analysis of the data revealed sparks with greater amplitude in left ventricle myocytes. The time for the calcium transient to reach half-maximum amplitude in the central part of the cell was found to be, on average, 2 ms shorter than at the cell ends. Sparks co-localized with t-tubules were found to have significantly longer duration, larger area and spark mass than those further away from t-tubules.

**Conclusion:** The high spatiotemporal resolution of the microscope and automated image-analysis enabled detailed 2D mapping and quantification of calcium dynamics of *n* = 60 myocytes, with the findings demonstrating multi-level spatial variation of calcium dynamics across the cell, supporting the dependence of synchrony and characteristics of calcium release on the underlying t-tubule structure.

## 1 Introduction

Under stable conditions, the cyclical contraction and relaxation of ventricular cardiomyocytes is regulated by calcium flow in and out of the cytosol. Influx of calcium ions into the cell occurs during activation of L-type calcium channels, located predominantly in the t-tubules, which have an average diameter of ∼250 nm and 1.8–2 μm periodicity along the long axis of the cell (Soeller and Cannell, 1999). The influx creates an increase in sub-sarcolemmal calcium concentration that results in activation of nearby type-2 ryanodine receptors (RyRs) (Bers, 2002)—calcium release channels on the sarcoplasmic reticulum (SR), typically grouped in clusters. Activated RyRs open, enabling rapid release of calcium from the SR into the cytosol. Localized release events from an activated cluster of RyRs, known as calcium sparks (Cheng et al., 1993), are confined to a diameter of ∼2 μm and occur on timescales of tens of ms (Cheng and Lederer, 2008). The cumulative effect of these events results in calcium transients, a global increase in calcium concentration throughout the cell, which initiates and sustains cell contraction.

Heart failure (HF) has been associated with structural changes in the organization and density of the t-tubule network (Lyon et al., 2009), resulting in “orphaned” RyRs (Song et al., 2006), which cannot be directly activated by surface Ca channels. Instead, the delayed activation of these orphaned RyR through Ca release from neighbouring clusters leads to reduced synchrony of Ca release (Louch et al., 2006), resulting in slower transients with lower amplitude and weaker contraction. These changes to cellular Ca control and the establishment of variable calcium dynamics within individual cardiomyocytes can lead to arrhythmia at the tissue level (Heinzel et al., 2011; Ibrahim et al., 2011). Previous studies have demonstrated that calcium spark frequency and the rising phase of calcium transients correlates with the proximity of calcium release sites and the t-tubule network (Dries et al., 2013; Sikkel et al., 2016), indicating that there may be differences between release events from coupled and orphaned RyR clusters. However, motion artefacts from cell contraction and limited spatiotemporal resolution of the employed imaging techniques have prevented multi-dimensional characterization of calcium transient development and calcium release correlation with cell structure.

Besides differences in calcium release in cardiomyocytes from healthy and pathological samples, the structural and functional heterogeneity of the heart has motivated the exploration of differences between the two ventricles at the cellular level. While left ventricle (LV) and right ventricle (RV) myocytes are known to have differences in structure and membrane organization, there have been limited and conflicting measurements of calcium handling and release in healthy cells isolated from left and right sides of the heart (Molina et al., 2016).

Studying the interrelationship of structural changes on the spatial variation and dyssynchrony of calcium release requires adequate sampling of the spatial features and events of interest across the cell. The correlative imaging of calcium sparks and transients with the cell microstructure requires acquisition rates on millisecond timescales, and sub-micron spatial resolution. While line scanning confocal microscopy has been extensively used for imaging calcium dynamics in cardiomyocytes (Shacklock et al., 1995; Louch et al., 2006; Song et al., 2006), the limited spatial information is inadequate for full characterization of calcium release in the physiologically anisotropic cardiomyocytes. Advances in scanning and detector technology have enabled high speed imaging at increased spatial dimensionality, allowing the visualisation of spatial variation in calcium release across the cardiomyocyte cell. For example, 2D confocal imaging at up 240 fps demonstrated a 2 μm periodic striation pattern in calcium indicator fluorescence at the start of the stimulated calcium transients in rat ventricular myocytes (Cleemann et al., 1998).

The challenges of achieving optically sectioned imaging with lower phototoxicity and fast acquisition speed are addressed by the fundamental principles of light-sheet fluorescence microscopy (LSFM), also known as selective plane illumination microscopy (SPIM) (Huisken et al., 2004), where illumination is confined to the imaged plane, enabling parallelized widefield acquisition of the fluorescence emitted from the illuminated slice. Oblique plane microscopy (OPM), a single objective LSFM technique, has been used for imaging calcium dynamics in cardiomyocytes at over 900 fps (Kumar et al., 2011). Dual-channel OPM has previously been used for correlating calcium release and dynamics with the sarcolemma and t-tubule network (Dries et al., 2013; Sikkel et al., 2016), with two-dimensional imaging at 667 fps showing increased spark frequency in tubulated regions, with higher spark rates at the tubules.

In this work, a custom light-sheet fluorescence microscope, described in (Sparks et al., 2020), is used for dual-channel 2D timelapse imaging of calcium and the sarcolemma, enabling sparks and transients in rat ventricular cardiomyocytes to be correlated with the cellular microstructure. The dual-objective LSFM geometry provides a higher detection NA and detection efficiency than the OPM system used previously, allowing the use of a lower average excitation power. Here, we implement a custom image processing pipeline for automated analysis of calcium dynamics from a total of 60 imaged cells. Automated detection and quantification of sparks and transients is used to resolve the spatial dyssynchrony of calcium release in healthy left and right ventricle cardiomyocytes, in correlation with the extracted t-tubule network map. Quantified parameters of spark and transient morphology are compared for LV and RV cardiomyocytes for further insight into physiological differences at the macro scale.

## 2 Materials and methods

### 2.1 Sample preparation

All studies were carried out with the approval of the local Imperial College London ethical review board and the Home Office, UK and in accordance with the Animals (Scientific Procedures) Act 1986 Amendment Regulations 2012, and EU directive 2010/63/EU, which conforms to the Guide for the Care and Use of Laboratory Animals published by the U.S. National Institutes of Health under assurance number A5634-01. The reporting in the manuscript follows the recommendations in the ARRIVE (Animal Research: Reporting of *In Vivo Experiments*) guidelines.

#### 2.1.1 Preparation of live dual-labelled ventricular cardiomyocytes

Left and right ventricular cardiomyocytes were enzymatically isolated from male rat hearts following the method outlined in (Sato et al., 2005) and then suspended in a low Ca^2+^ enzyme solution containing (in mM): NaCl (120), KCl (5), MgSO_4_ (5), Na pyruvate (5), glucose (5), taurine (20), HEPES (10), CaCl_2_ (0.2), pH 7.4 adjusted with 1 M NaOH. The cells were stored at room temperature and imaged within 8 h of isolation. The live-cell dual labelling preparation protocol was adapted from that used for OPM-based imaging of live cardiomyocytes (Sikkel et al., 2016), and our proof-of-concept live cell LSFM imaging (Sparks et al., 2020). Simultaneous monitoring of calcium dynamics and intracellular tubular microstructure was achieved by labelling the isolated adult rat cardiomyocytes with cell-permeant calcium indicator Fluo-4 AM (ThermoFisher Scientific) and the plasma membrane stain CellMask Orange (CMO) (ThermoFisher Scientific). For each labelling procedure, 1 mL of the myocyte suspension from each ventricle was incubated with 0.16% pluronic acid and 5 µM Fluo4-AM (dissolved in dimethyl sulfoxide, DMSO) for 15 min at 37°C, followed by the addition of 5 μg mL^−1^ CMO, and incubated for another 5 min. Homogenous dye distribution during incubation was achieved by placing the cell suspension on a rotary mixer, and light exposure was minimized by wrapping the sample tube in aluminium foil.

After the incubation time, the cells were centrifuged at low speed [21 RCF (relative centrifugal force, or G-force)] at 20°C for 1 min to form a pellet. The supernatant was removed and the pellet was resuspended in a “working solution” composed of a 1:1 mixture of Dulbecco’s modified Eagle’s medium (DMEM) (Gibco BRL, Life Technologies Ltd.) solution containing 1.8 mM Ca^2+^ and enzyme solution containing 0.2 mM Ca^2+^ and left in the dark at room temperature for 30 min for de-esterification. For imaging, the cells were attached to the bottom of the imaging chamber using 1.5 µL mouse laminin (Gibco) and immersed in Normal Tyrode (NT) containing (in mM): NaCl (140), KCl (6), glucose (10), HEPES (10), MgCl_2_ (1), CaCl_2_ (1), pH adjusted to 7.4 with NaOH. To avoid motion artefacts during cell contraction and enable correlative mapping of calcium dynamics with cell structure, para-nitroblebbistatin (NBleb) (Axol Bioscience), a non-phototoxic low-fluorescence derivative of the myosin inhibitor blebbistatin (Képiró et al., 2014) was used to decouple cell contraction from the calcium dynamics. The decoupler was added to the “working solution” and to NT at 25 µM concentration.

### 2.2 Data acquisition

#### 2.2.1 Dual-channel light sheet fluorescence microscope

The design and operation of the dual-channel SPIM system has been described in detail in our previous work (Sparks et al., 2020), and this system was used with minor modification. Briefly, angularly dithered Gaussian light-sheet illumination is used to excite sample fluorescence at a 37-degree angle relative to the horizontal surface. The light-sheet is generated by focusing the laser excitation to a line in the back focal plane of the water immersion 10× 0.3 NA illumination objective.

The emitted fluorescence is collected by a 1.0 NA 20× water immersion objective orthogonal to the light-sheet plane. The detection plane is imaged onto a two-dimensional adjustable slit that defines the FOV boundary, and reimaged onto a sCMOS detector, at a sample-to-image magnification of 44×. The remote-refocusing elements described in the previous paper (Sparks et al., 2020) were bypassed by replacing the polarizing beam splitter cube with a 100% mirror. Simultaneous dual-channel imaging of the Fluo-4 and CMO emission is achieved by spectrally separating the fluorescence using a 560 nm edge long-pass dichroic beam splitter and 525/50 nm (centre/bandpass) and 630/75 nm emission filters. The two spectral channels are laterally displaced onto separate halves of the camera sensor using positioning mirrors, with the FOV corresponding to each channel aligned such that their long axes were adjacent to each other and perpendicular to the camera sensor’s row-by-row readout direction.

Longitudinal chromatic aberration in the optical system gave rise to a ∼10 mm axial separation at the camera between the image foci of the two spectral channels. To correct for this, two doublets of near-equal but opposite focal length (160 mm and −150 mm) were inserted in the Fluo-4 emission path, with principal planes separated by ∼15 mm, which introduced 0.9× magnification of the Fluo-4 spectral channel compared to the CMO channel (Dvinskikh, 2022).

#### 2.2.2 LSFM imaging of live left and right ventricle cardiomyocytes

Fluo-4 and CMO were excited simultaneously by the 488 nm and 561 nm laser lines at 25 µW and up to 35 µW power respectively, measured in the back focal plane of the excitation objective. Two-dimensional dual-channel time-lapse data of the fluorescence emission was acquired using a Hamamatsu Orca Fusion sCMOS camera with a 6.5 μm × 6.5 μm pixel size. The camera was operated in normal area external trigger mode, with synchronous readout trigger at 395 fps for a 1,152 × 512 pixel region of interest (ROI), which corresponds to a 170.2 × 75.6 μm FOV in sample space at 44× magnification and 0.1477 μm pixel size.

The horizontal extent of the camera ROI was chosen to fit the length of a cardiomyocyte image, and the height was set to fit two vertically displaced spectral channels, each large enough to fit the width of a cardiomyocyte image. Sample navigation was carried out using a widefield low-NA objective mounted below the sample stage in transillumination, allowing selection of cells which had their long axis approximately perpendicular to the light-sheet propagation direction. This enabled faster acquisition of a longitudinal orthogonal slice image of the cell, illuminated with the thinnest part of the light-sheet. The acquisition duration ranged between 12,000 and 18,000 frames (∼30–45 s). The cells were electrically stimulated starting 1 min before each acquisition, with the electrical stimulation maintained for the first half of each acquisition. The synchronization monitor signal from the electric field stimulation unit was recorded as a digital input into a digital acquisition card connected to the computer, controlling the image acquisition.

Cardiomyocytes isolated from left and right ventricles of each male rat heart were imaged on the same day. Image acquisition and analysis on each day was performed blinded with respect to the two ventricles, labelled as “A” and “B.” The imaging was performed in two batches with separate dye loading procedures, each containing an aliquot from each ventricle (cells from “A” then cells from “B,” or *vice versa*), with the imaging order reversed for the second load.

The imaging chamber for single adult cardiomyocytes consisted of a glass slide, with a 2 mm high silicone strip around the perimeter to contain the immersion liquid (Supplementary Figure S1). Electric field stimulation at a frequency of 0.5 Hz and 2 ms pulse duration was delivered by 0.8 mm diameter platinum electrodes separated by 5 mm at 1.5× the threshold voltage for contraction, which was found to be approximately 20 V.

### 2.3 Data processing and image analysis

#### 2.3.1 Pre-processing

All acquisitions were previewed in ImageJ software, with intensity time traces taken through rectangular ROIs placed manually within the cell images to provide an overview of the calcium dynamics in the cell. Each acquisition was screened based on the following four criteria to determine whether it would be included for subsequent analysis: 1) cell health, 2) successful decoupling, 3) transient presence and amplitude and 4) acquisition quality (Supplementary Note S1.2). All subsequent image analysis was implemented in MATLAB.

The acquired x-y-t data underwent a series of pre-processing steps, including spectral channel co-registration, background subtraction and segmentation (Supplementary Note S1.3). The resultant cell mask (Supplementary Figure S2D) was used to define the cell area for the next steps of the analysis. A co-registered and merged channel time-lapse acquisition is demonstrated in Supplementary Video S1.

#### 2.3.2 Identifying the t-tubule structure and nuclei

Spatial correlation of calcium dynamics with the t-tubule network requires a method of extracting the structural features from the cell image. Several existing methods such as the MATLAB-based AutoTT (Guo and Song, 2014), the ImageJ/Fiji plugin TTorg (Pasqualin et al., 2015), and the method used in (Sikkel et al., 2016) identify the tubule map by utilizing the network periodicity, extracting the spatial frequency peaks from the two-dimensional Fourier transform of the image. However, these methods use the frequency domain to treat only transverse or longitudinal tubules, excluding elements of a different alignment to the cell’s longer axis. In our approach, the structural features were determined without any prior assumptions of their orientation, and the analysis was carried out directly in image space. The binary tubule mask was generated by intensity thresholding of a filtered average taken across all frames of the CMO channel for each cell, and the nuclear segmentation was achieved through binary masking of manually outlined regions of the averaged CMO image for each cell (Supplementary Note S1.4).

Certain cells did not have an identifiable nucleus in the plane imaged, while others had up to two visible nuclei. The t-tubule period, measured from a line profile through a tubulated region, was estimated to be 2 µm (or 14 pixels, to the precision of one pixel), in agreement with measurements in other studies (Soeller and Cannell, 1999). A selection of t-tubule maps with masked nuclei from 8 ventricular cardiomyocytes is shown in Supplementary Figure S4, with the full set of maps for all analyzed cells (*n* = 60) presented in Supplementary Figure S5. In total, 48 nuclei were labelled, with nuclei identified within the imaged plane in 40 out of the 60 cells analyzed.

#### 2.3.3 Distance to nearest tubule

**FIGURE 1:**
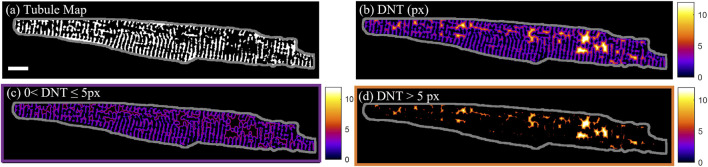
T-tubule and distance to nearest tubule maps. **(A)** Binary tubule map of cell D with the cell outline shown in grey. **(B)** Pixel distance to nearest tubule (DNT) map of cell D. **(C)** Tubulated regions of cell D with 0 < DNT ≤ 5 px. **(D)** De-tubulated regions of cell D with DNT > 5 px. Scalebar: 10 µm.

For quantification of the distance of calcium dynamics to the cell structure, the distance of the calcium release events to the closest part of the tubular network was assessed. The “distance to the nearest tubule” (DNT) map was calculated by taking the Euclidian distance transform (Maurer et al., 2003) of the tubule binary mask. In Figure 1, this is demonstrated on cell D from Supplementary Figure S4, which has less than the average tubule coverage of the cell area (34% in comparison to the 39% ± 4% (mean ± SD) across *n* = 60 cells. The DNT metric was used to distinguish between tubules (DNT = 0), tubulated (0 < DNT ≤ 5 px) and detubulated (DNT > 5 px) regions of the cell (Figures 1B–D). The 5-pixel (0.74 µm) threshold used to distinguish tubulated and detubulated areas was determined from the largest DNT value present in line profiles through visually identified areas of well-organized tubules.

#### 2.3.4 Spark analysis

##### 2.3.4.1 Spark detection

Spark detection and characterization was performed using a custom MATLAB code adapted from an earlier version implemented for the detection and characterization of sparks in OPM 2D time-lapse data (Maioli, 2016; Sikkel et al., 2016). Calcium spark analysis was performed on the last 5,000 frames (12.7 s) of each acquisition during which the electrical stimulation was switched off. Spatio-temporal smoothing was applied by convolving the x-y-t data with a 5 × 5 × 3 kernel. To account for any temporal variation in cell background intensity, the 5,000-frame dataset was divided into 5 × 1,000 frame intervals over which the background was assumed to be constant with time. To normalize for varying labelling levels and baseline fluorescence between individual cells, the spark detection algorithm was implemented on data divided by the mean intensity map, calculated for each 1,000 frame interval.

The initial detected sparks were identified as connected components with intensities above the lower threshold *T*_*L*_, with at least one point above a higher threshold *T*_*H*_. The selected thresholds were defined as *T*_*L*_ = *µ* + 2.9*σ* and *T*_*H*_ = *µ* + 5.4*σ*, where *µ* is the mean and *σ* is the standard deviation in Δ*F*/*F*_0_ for each pixel across each 1,000-frame interval, where Δ*F* = *F* − *F*_0_ and *F*_0_ is the baseline cell fluorescence. Any sparks present during each interval will influence the estimates of cell baseline fluorescence, and hence to improve the spark detection accuracy, the algorithm was run twice. After the first iteration, the detected preliminary sparks were masked out, and the baseline fluorescence *F*_0_ was recalculated across the first 100 frames of each analyzed data segment. The spark detection algorithm was repeated with the recalculated mean and standard deviation x-y maps with the preliminary sparks excluded. Final sparks were identified as connected components in x-y-t with a minimum 6-pixel connectivity.

##### 2.3.4.2 Spark characterization

The x-y-t spark mask was collapsed along the time axis to give a 2D spark mask which included any pixel that reached intensity values above the threshold *T*_*L*_ during the spark duration. The *spark area* was calculated as the total number of pixels within the 2D x-y spark mask. Next, the two-dimensional spark mask was projected along the time axis and the temporal intensity profile was found by averaging over the spark area in x-y. The *spark amplitude* (Δ*F*/*F*_0_) was calculated as the difference between peak and baseline intensity divided by the latter, and the *spark full-duration half-maximum (FDHM)* was defined as the time between the first and last time points that the spark is above half of its peak intensity above baseline (F50). The *centre of mass (COM)* coordinates in x and y were calculated by taking the weighted average of the spark projections profiles in the two spatial directions and defined the spark location within the cell. Additionally, the *spark FWHM* in *x* and *y* directions were calculated by taking line profiles through the spark peak in time and centre of mass coordinate and counting the number of pixels with intensities at or above F50. The *calcium spark mass* was defined as the product of the spark amplitude, FWHM and FDHM: Mass = Δ*F*/*F*_0_ × FWHM × FDHM, with the FWHM calculated as the average of the respective FWHM in *x* and *y* directions, extending the definition used by (Kolstad et al., 2018) with line-scan confocal to 2D time-lapse data.

**FIGURE 2:**
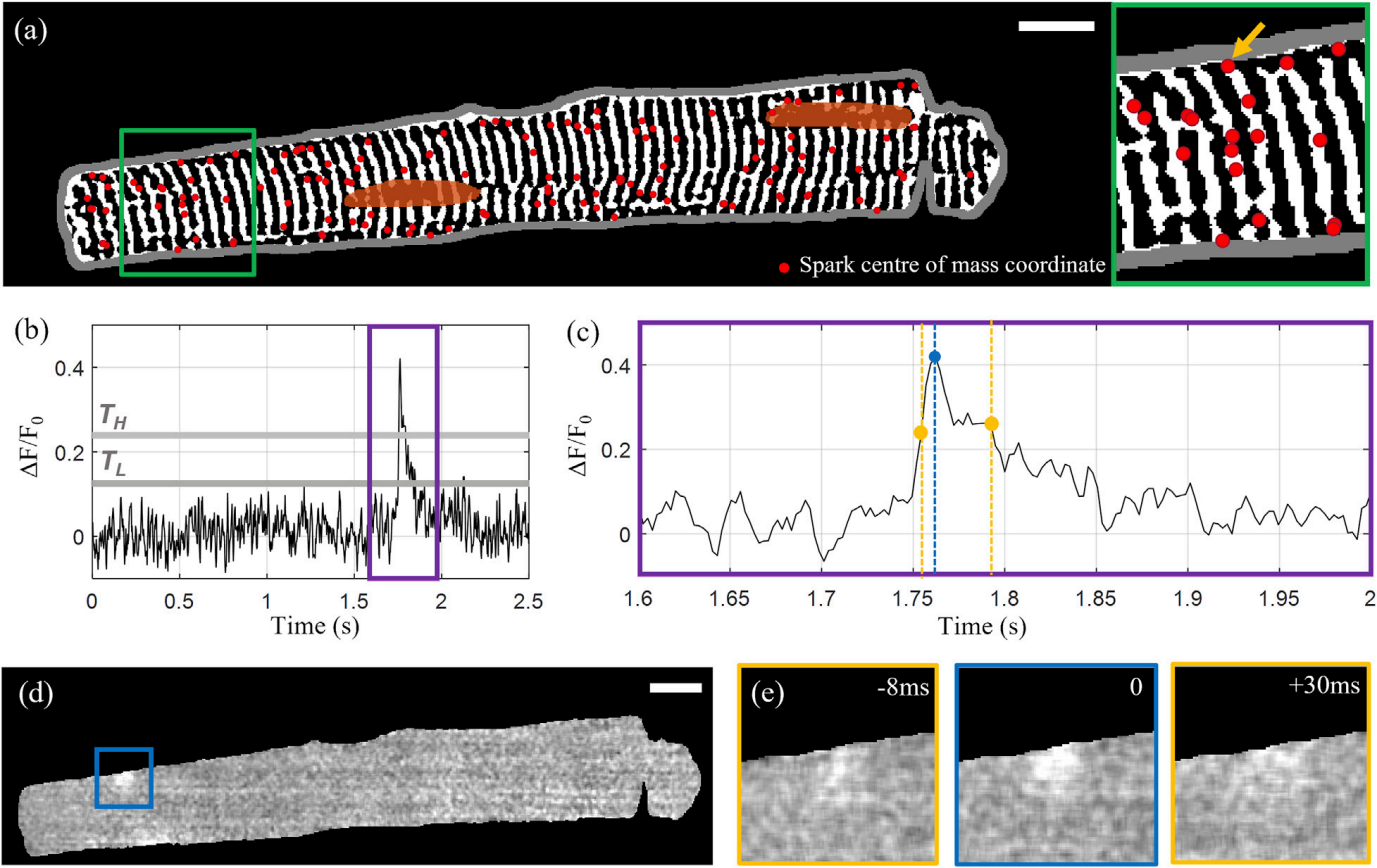
Calcium spark detection and correlation with cell structure. **(A)** Centre of mass coordinates (red markers) for a total of 139 detected sparks overlaid on the extracted tubular microstructure of the example Cell X. The cell membrane outline is shown in grey and the manually segmented nuclei are shown in orange. **(B)** Intensity time profile through the 1,000-frame sequence for the spark indicated by the yellow arrow in the inset shown on the right side of **(A)**. The upper and lower intensity thresholds are indicated by *T*_*H*_ and *T*_*L*_ respectively (grey lines). **(C)** A zoom-in of the time region indicated by the purple rectangle in **(B)**. The detected peak is indicated by the blue marker, and the first and last frames above F50 are indicated with yellow. **(D)** Normalized fluorescence intensity at the time point corresponding to the selected spark’s peak intensity. **(E)** The 100 by 100 pixel region centred on the spark centroid indicated by the blue square in panel **(D)** at peak intensity (middle) with the intensity distributions at the timepoints corresponding to F50 before and after the peak shown on the left and right respectively. Scalebar: 10 μm.

The identified sparks were filtered by area and duration to exclude the false detection of events arising as a consequence of noise. Sparks with areas below 50 pixels (<1 μm^2^) and durations (here defined as time the spark is “on” or above the lower threshold T_L_) below four frames (10 ms) were rejected. “Unfinished” sparks that did not fall below F50 within the time series were also excluded. The centre-of-mass coordinates of the 139 detected sparks overlaid with the extracted t-tubule structure for the example Cell X are shown in Figure 2A, with Figures 2B–E illustrating the rise and decay of one of the sparks in x-y-t dimensions.

#### 2.3.5 Transient time-to-half-maximum

Some previous studies (Mukamel et al., 2009; Amanfu et al., 2011) and tools such as CalTrack (Psaras et al., 2021) achieve automated quantification of calcium transient dynamics by averaging intensity time traces across all pixels within a segmented cell, which does not offer insight into the sub-cellular spatial variation of calcium release. In our approach, we achieve automated analysis of the spatial dyssynchrony of calcium transient development by determining T50—the time point when the calcium transient reaches half-peak intensity (F50) for each pixel. This time point was determined through pixel-wise calculation of peak and baseline Fluo-4 emission intensity levels, and linearly interpolating the time point corresponding to F50.

##### 2.3.5.1 Pixel-wise determination of transient time-to-half-maximum (T50)

The calcium transient analysis was performed on an 8 s (3,160 frame) sequence from the paced part (first half) of each acquisition, corresponding to four pacing periods, starting two pacing periods (4 s/790 frames) in. The x-y-t data set was smoothed spatially using a 5 px × 5 px median filter and temporally using a moving average over a 3-frame window. The segmented cell mask (Supplementary Figure S2D) was applied to the transient data to define the cell area. The global cell transient peaks along the time dimension were identified with the minimal peak separation time set to 95% of a single pacing period.

**FIGURE 3:**
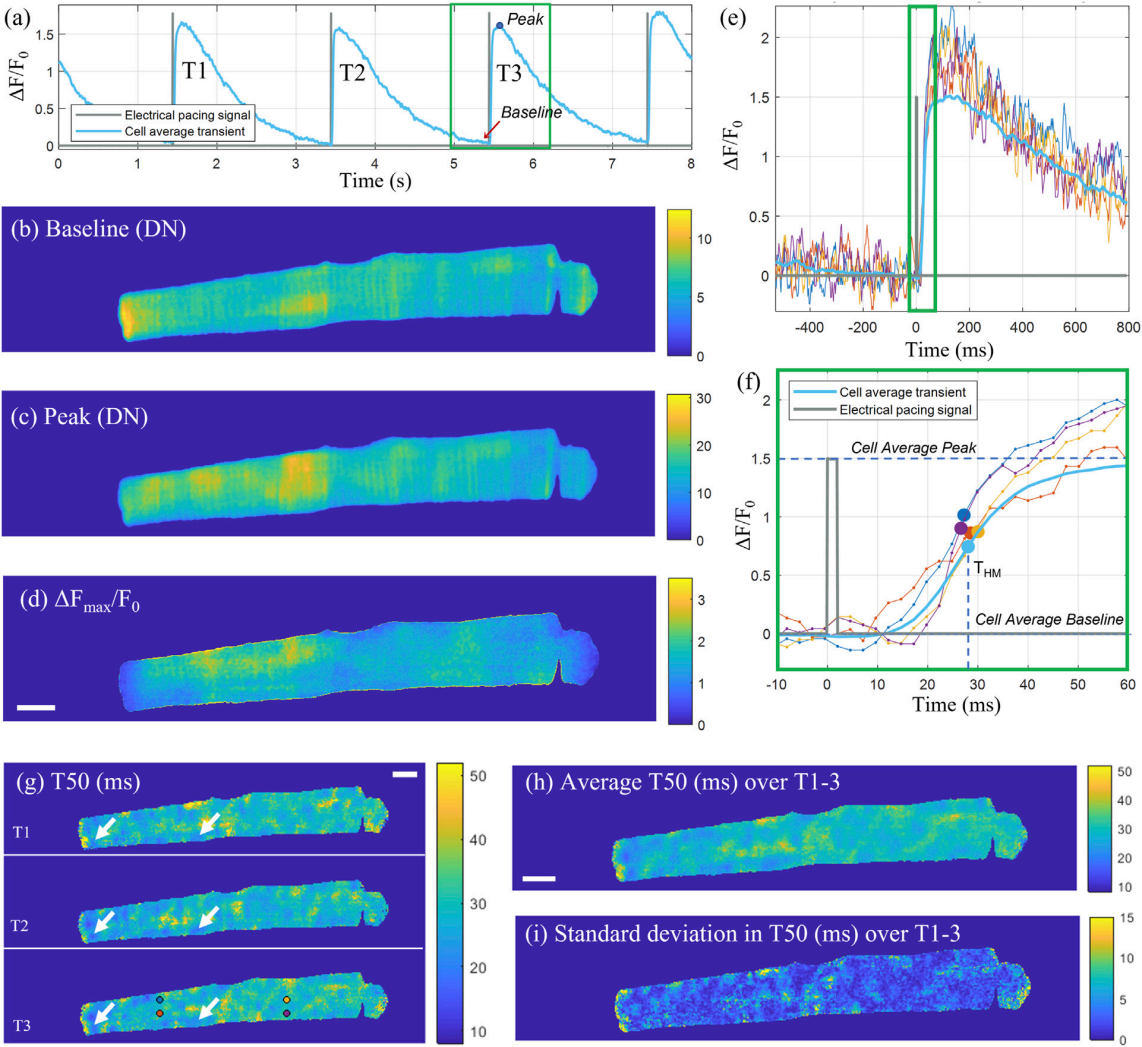
Pixel-wise determination of transient T50. **(A)** Cell average transient (ΔF/F_0_) for the example cell X as a function of time across the sampled time-sequence including three consecutive transients (T1, T2, T3). The recorded pacing signal at 0.5 Hz is shown in grey. For the third transient T3, cropped to a 525 frame (1.3 s) window (green rectangle), two-dimensional maps for the baseline fluorescence (in digital numbers), peak intensity (in digital numbers) and Δ*F*_max_/*F*_o_ are shown in **(B–D)** respectively. **(E)** Intensity time traces through individual pixels indicated with circular markers in panel **(G)** for T3, and the cell-average time trace for T3. **(F)** Zoom-in on the rising edge of the transient [indicated by the green rectangle in **(E)**] with the *T*_HM_ time points for the selected pixel traces indicated with markers. **(G)** T50 (ms) maps for the three consecutive transients, with some exemplar repeating features, corresponding to areas of early T50, indicated by white arrows. **(H)** Average T50 map (ms) and **(I)** standard deviation in T50 (ms) of the three transients. Scalebar: 10 μm.

A total of three consecutive transients were selected for analysis, with each individual transient cropped to a 525 frame (1.3 s) window centred on the peak (Figure 3A). The electrical pacing trace was cropped to the same time window as the transient, and the rising edge of the top-hat shaped pulse, was used as the excitation start time and a consistent point of reference for the transient onset. The time-to-half-maximum (T50) was defined as the time from the start of the pacing signal until the transient reaches half of its peak amplitude (F50) for that pixel, where F50 is defined as half of the difference between the peak and baseline intensity for that pixel. The baseline intensity (Figure 3B) was calculated as the average intensity across a 50-frame window (126 ms) up until the start of the pacing signal. The peak intensity (Figure 3C) for each pixel was calculated as an average across 20 frames starting from the cell average peak intensity time point. The ΔF/F_0_ signal was calculated by dividing the pixel-wise difference between the signal and the baseline by the baseline, with the corresponding map at the transient peak shown in Figure 3D. After calculating the half-max intensity, the corresponding time point was identified by linearly interpolating between the last frame before and first frame after F50 (Figures 3E, F). The time-to-half-maximum was then calculated as the duration from the electrical excitation reference time point until the time point of F50. The T50 maps for the three consecutive transients are shown in Figure 3G, with some repeated features, corresponding to spatial domains with earlier transient T50 compared to the rest of the cell, consistent across the three transients (indicated by white arrows). The average and standard deviations of the T50 maps for three consecutive transients are shown in Figures 3H, I respectively.

For the cell displayed in Figure 3, the median ± inter-quartile range (IQR) time-to-half-maximum for the map representing the cell average across three transients was 28.9 ± 4.6 ms. The automated analysis was applied to all 60 cells, with a selection of T50 maps for eight of the cells shown in Supplementary Figure S6, with the rest of the resultant T50 maps shown in Supplementary Figure S7. The time-to-half-maximum across all analyzed cells, calculated as an average over the medians for each T50 distribution for each cell, was equal to 29.35 ± 4.62 ms (mean ± SD), with a standard error of SE = SD/√*n* = 0.60 ms.

Upon initial inspection of the T50 maps, a range of features could be observed. Firstly, the T50 distribution was spatially non-homogenous across a (healthy) cell, with a “patchy” pattern visible. The ends of multiple cells (Supplementary Figures S6A–C, E, G) had visibly lower T50 values, indicating earlier transient rise. We could also observe delayed T50 in nuclear regions (cells B, C, G, H), and a transverse striped pattern resemblant of the t-tubule network visible in certain regions (cells A, B, C, G). To evaluate the dyssynchrony in calcium transients across the cell, these features were investigated quantitatively by comparing the T50 for the outer and central areas of the cell, as well as T50 in nuclear regions compared to the rest of the cell, and correlating the T50 spatial variation with the cell structure.

The nuclear and exonuclear T50 were calculated as the median time-to-half-maximum across all pixels within the nuclear mask and pixels outside the nuclear mask respectively. To compare the time-to-half-maximum at the ends of the cell to the middle, assuming approximate alignment of the cell with the horizontal axis of the FOV, the cell was divided into rectangular regions along its length. The two ends of the cell were identified from the first and last horizontal pixel coordinates corresponding to a non-zero value within the (masked) T50 map. Next, the cell was divided into three regions (indicated by the magenta rectangles in Supplementary Figure S6, cell C): a central ROI with the longer side corresponding to half of the cell length, and two ROIs at each end of the cell with the horizontal dimensions of both ROIs equal to a quarter of the cell’s length. The outer ROIs were grouped together, and the area corresponding to the segmented nuclei was excluded.

### 2.4 Statistical analysis

For each parameter, the D’Agostino-Pearson normality test (D’Agostino and Pearson, 1973) was used to test for a normal (Gaussian) distribution, and, if the dataset did not pass the normality test, for lognormal distribution, to evaluate whether parametric testing should be applied. For normal and log-transformed lognormal distributions, the paired and unpaired *t*-tests (PT and UT respectively) were used to determine statistical significance. When applying two-sample *t*-tests, the *F*-test of equality of variances was used to test for homoscedasticity, or the homogeneity of variance across the datasets. When the variances of two datasets were significantly different, the unpaired *t*-test was applied with Welch’s correction (UTWC) (Welch, 1947). For datasets which did not follow a normal or log-normal distribution, the unpaired Mann-Whitney test (MW) (Mann and Whitney, 1947) and paired Wilcoxon signed rank test (WSRT) (Wilcoxon, 1946) were used. Statistical analysis of significance was carried out in GraphPad Prism 9.1.1 software. *p*-values < 0.05 were considered statistically significant.

## 3 Results

### 3.1 Sparks morphology and correlation with t-tubule structure

A total of *n* = 60 acquisitions from unique cells were analyzed from seven different male rat hearts, with 5,785 sparks detected across the 60 cells, with an average of 96 ± 66 sparks (mean ± SD) per analyzed 12.7 s sequence per cell. The calculated spark amplitude, area, duration, and mass distributions across all sparks are summarized in histograms in Supplementary Figure S8. The modal distribution of the spark amplitude agrees with previous findings of a preferred amplitude for in-focus sparks from x-y-z-t data acquired with fast-scanning confocal (Shkryl et al., 2012) rather than the monotonically decaying amplitude distribution observed through confocal line scan imaging (Cheng et al., 1999).

The average distance from a randomly chosen pixel to the nearest detected tubule was 1.5 ± 0.5 pixels (mean ± SD, standard error SE = SD/√*n* = 0.06), while the average spark centre of mass (COM) distance to nearest tubule (DNT) was equal to 0.85 ± 0.5 pixels (mean ± SD, SE = 0.07), indicating that the spark COM are significantly (*p* < 0.0001, MW and WSRT test) more likely to be located at a t-tubule than a random pixel in the cell. Supplementary Table S1 summarizes the total number of sparks (average per cell), grouped in three different spatial categories based on their DNT: epitubular (DNT = 0), paratubular (0 < DNT ≤ 5 px) and sparks from detubulated regions (DNT > 5 px). On average across all cells, 63% of sparks per cell had a COM co-localized with the tubules. A further 30% sparks per cell are within 3 pixels (0.44 µm) of tubules, and a further 5% are within 5 pixels (0.74 µm). Only 1.6% of sparks per cell are found to be more than 5 pixels (0.74 µm) away from the nearest tubule. Only 6 out of 60 cells had more than three sparks in detubulated regions, and hence sparks from these areas could not be statistically compared with sparks from other spatial categories.

**FIGURE 4:**
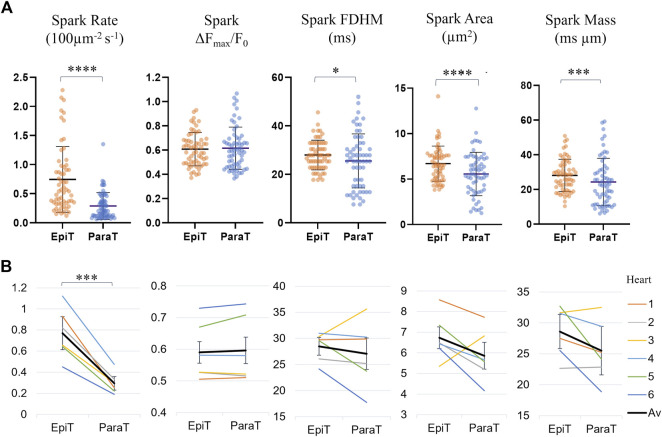
Spark parameters compared for epitubular (EpiT, DNT = 0) and paratubular (ParaT, 0 < DNT ≤ 5) regions. **(A)** Spark parameter distributions, with each datapoint corresponding to the median across epitubular (orange) and paratubular (blue) spark populations within each cell. The horizontal lines indicate the mean ± SD over all cells (*n* = 60). The levels of significance illustrated in the form of stars are based on paired testing (see Supplementary Table S2). **(B)** Median spark parameter for epitubular and paratubular spark populations in each cell, averaged for each heart (1–6, see legend). The overall average (“Av”) across the six hearts is shown in black. The error bars represent the standard deviation across the heart averages. When comparing cell-by-cell differences, averaged for each heart using a one-sample *t*-test, only the spark rate is significantly different between epitubular and paratubular populations.

Supplementary Table S2 and Figure 4 compare the calculated spark parameters for epitubular and paratubular sparks. Comparing the average across the means calculated for all sparks within each DNT category for each cell using paired testing, epitubular sparks were found to have 8% longer FDHM (28.0 ± 6.1 ms vs. 25.5 ± 11.1 ms, *p* = 0.016, PT), 18% larger area (6.70 ± 1.91 µm^2^ vs. 5.51 ± 2.34 µm^2^, *p* < 0.0001, WSRT), and 13% larger spark mass (28.1 ± 1.2 ms µm vs. 24.3 ± 13.6 ms µm, *p* = 0.0002, PT) than paratubular sparks. Accounting for the area of the cell belonging in each spatial DNT category, the spark rate, (number of sparks per 100 μm^2^ per second) in paratubular regions was ∼60% lower than in epitubular regions (*p* < 0.0001). The difference in spark amplitude between epitubular and paratubular sparks was not significant (0.61 ± 0.14 vs. 0.62 ± 0.17, *p* = 0.92, PT).

Figure 4B shows the spark parameters for epitubular and paratubular spark populations in each cell, averaged over all cells from each isolation. One of the *N* = 7 isolations, from which only one cell was included in the final analysis, was excluded from the heart averages presented in all following results. For the remaining heart isolations, the number of cells analyzed varied between 6 and 14. When applying a one sample *t*-test to the differences for spark parameters in each cell, averaged for all cells from each heart, only the spark rate was significantly different between paratubular and epitubular spark populations.

### 3.2 Transient dyssynchrony and correlation with t-tubule structure

**FIGURE 5:**
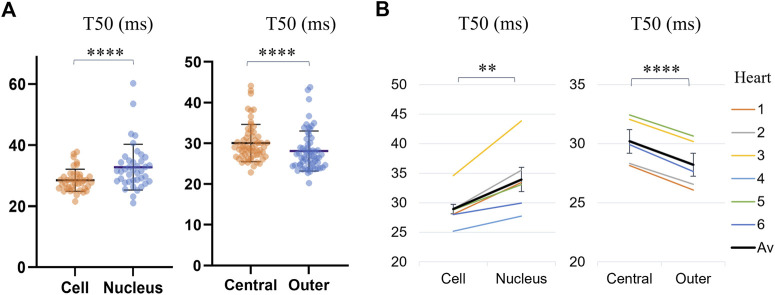
Comparison of average time-to-half-maximum (T50) for different regions of the cell: the cell (excluding the nucleus), the nucleus, the central IQR and the outer quarters. **(A)** Transient T50 distributions, with each datapoint corresponding to the median across all pixels within a region of a cell. The horizontal lines indicate the mean ± SD over all cells (*n* = 40 for cells with identified nuclei for the first category, and all *n* = 60 cells for the second category. Levels of significance illustrated with stars are based on paired testing (see Supplementary Table S3). **(B)** Transient T50 in different regions of each cell, averaged over the cells from each heart (1–6, see legend). The overall average (“Av”) across the six hearts is shown in black. The error bars represent the standard errors (SE = SD/√n) across the heart averages. When comparing cell-by-cell differences, averaged for each heart using a one-sample *t*-test, both comparisons maintained statistical significance, with the nuclei and central regions of the cell having a significantly longer time-to-half-maximum than the rest of the cell and the outer region respectively.

The T50 values for different regions of the cell, across all analyzed cells are presented in Supplementary Table S3 and Figure 5. Transients in the nuclei were found to reach F50 later than the rest of the cell by Δ = 3.47 ms on average (*p* < 0.0001, WSRT, for *n* = 40 cells with detected nuclei). By dividing the cell into central and outer regions, it was found that, on average, the outer regions of the cells reached F50 by Δ = 1.97 ms earlier than the cell middle (*p* = 0.0018, WSRT, for *n* = 60 cells), indicating further spatial dyssynchrony of calcium transients across the cell.

**FIGURE 6:**
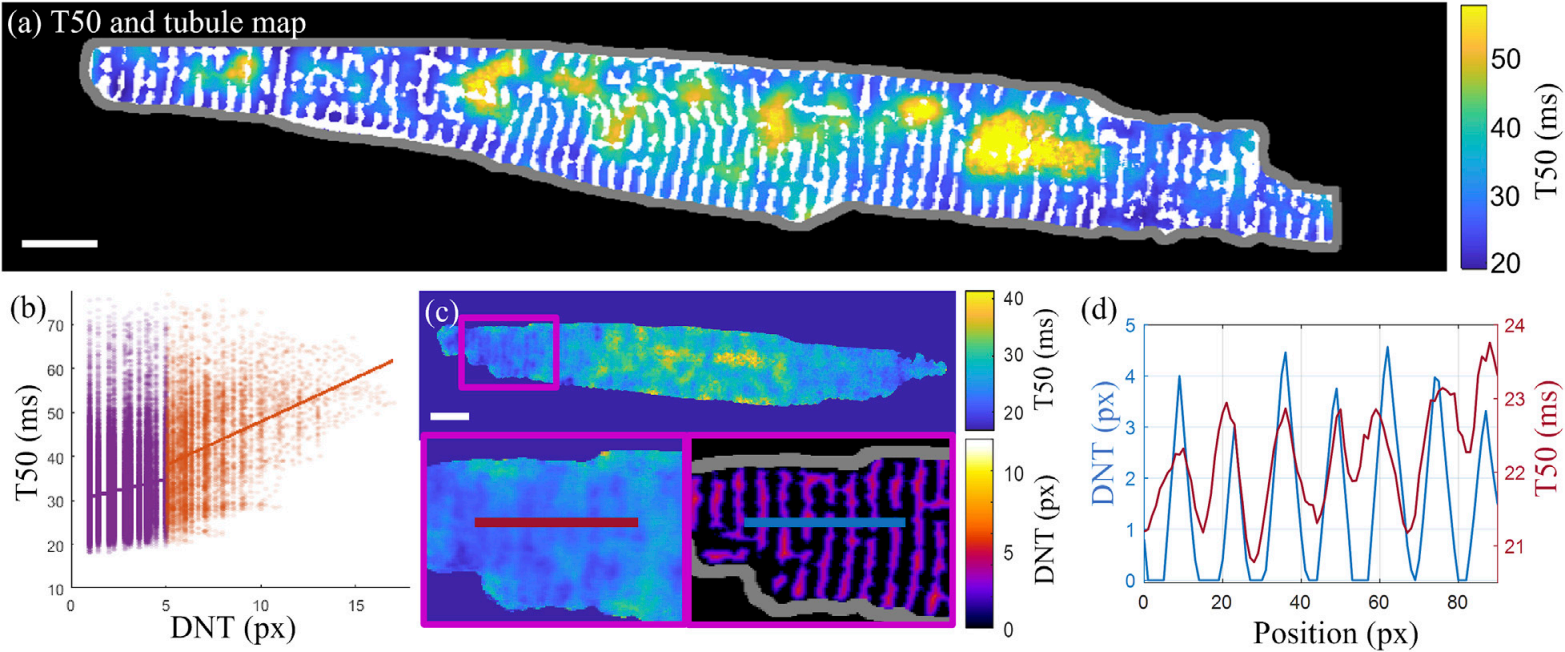
Correlation of the t-tubule structure with transient time-to-half-maximum **(A)** Time-to-half-maximum (T50, in ms) map for the T50 average over three consecutive transients of cell D with binary t-tubules overlaid in white. **(B)** T50 correlation with DNT across all pixels in cell **(D)**. Linear fits to tubulated (purple) and detubulated (orange) regions have slopes of 0.99 ms/px, 95% CI [0.94 1.04], and 1.99 ms/px, 95% CI [1.89 2.08], indicating increased positive correlation of T50 with DNT in detubulated regions. **(C)** Horizontal line profiles (averaged vertically over a 5-pixel width) taken through the T50 (left and top) and DNT (bottom right) maps of cell C. **(D)** The line profiles through T50 and DNT maps shown in panel **(C)** are overlaid, indicating earlier transient rise in areas closer to tubules. Scalebar: 10 µm.

Overlaying the T50 map with the tubule structure demonstrates how some cell regions with delayed transient rise (with larger T50 values) coincide with cell areas that lack regular tubule structure (Figure 6A). This pattern was quantified in terms of the slope of the linear fit to the correlation scatterplots of T50 and DNT values for each pixel in the cell (Figure 6B). For the example cell shown in Figure 6A, the slopes are 0.99 ms/px, 95% CI [0.94 1.04], and 1.99 ms/px, 95% CI [1.89 2.08], for tubulated and detubulated regions respectively, indicating positive correlation of T50 with DNT, with a stronger correlation in detubulated regions. Taking a horizontal line profile through the T50 and DNT maps through a tubulated region in cell C (Figures 6C, D) indicates delayed transient development in between the tubules.

**FIGURE 7:**
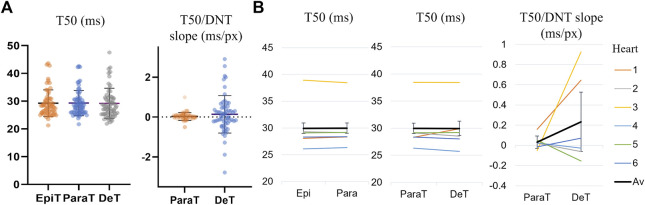
Transient T50 and T50/DNT slope for different DNT regions. **(A)** Transient T50 distributions, with each datapoint corresponding to the median T50 (ms) across all pixels within epitubular (EpiT, DNT = 0), paratubular (ParaT, 0 < DNT ≤ 5) or detubulated (DeT, DNT > 5) regions. The horizontal lines indicate the mean ± SD over all cells (*n* = 60). None of the differences were statistically significant. **(B)** Transient T50/DNT slope in different regions of each cell, averaged over the cells from each heart (1–6, see legend). The overall average (“Av”) across the six hearts is shown in black. The error bars represent the standard errors (SE = SD/√n) across the heart averages. When comparing cell-by-cell differences, averaged for each heart using a one-sample *t*-test, there was no significant difference in T50 and T50/DNT slopes in parts of the cell corresponding to the various DNT categories.

When considering the median T50 values within each DNT category averaged across all cells, there was no significant difference between the time-to-half-maximum within epitubular (DNT = 0), paratubular (0 < DNT ≤ 5) or detubulated (DNT > 5) regions (Figure 7; Supplementary Table S4). The average slope of the linear fits to the transient T50 and DNT correlation scatterplots in each cell was 0.03 ± 0.20 ms/px (mean ± SD), 95% CI [0.00 0.06] and 0.14 ± 0.93 ms/px (mean ± SD), 95% CI [-0.11 0.38] for tubulated and detubulated regions respectively, with no statistically significant difference between the two regions (Figure 7; Supplementary Table S5).

### 3.3 Comparison of left and right ventricle cardiomyocytes

All image acquisition, data selection, processing and image analysis was performed blinded with respect to ventricle. However, upon unblinding, it was revealed that the labelling of the received batches of cells for each ventricle (“A” and “B”) had not been randomized on each acquisition day. While the ventricle imaging order for the two loads on each acquisition day had been alternated, it was not randomized, resulting in more RV CM imaged first in the sequential acquisition process. The calculated parameters for the full dataset are presented in Supplementary Table S6—with no significant differences found between myocytes from the two ventricles.

To evaluate the possibility of the time since start of loading having a systematic influence on the measured parameters, their correlation was assessed (Supplementary Note S2.3; Supplementary Table S7; Supplementary Figure S9). To reduce the possibility of systematic bias due to imaging time point on differences in the parameter comparison between the two ventricles, the comparison reported below was performed on a subset of the data that only included cells imaged within a shorter 40-min time window (Supplementary Figure S9). The window was selected to maximize imaging time point range overlap for both ventricles in order to reduce any potential bias. This resulted in a sample size reduction to *n* = 24 cells.

**FIGURE 8:**
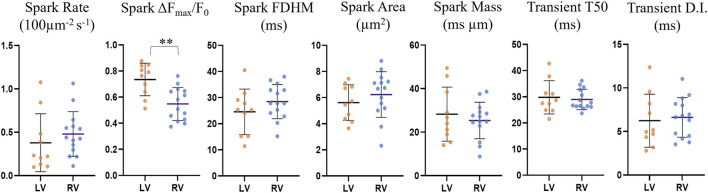
Comparison of spark and transient parameters for LV and RV cardiomyocytes for the restricted *n* = 24 dataset. The horizontal lines represent the averages across the medians for each cell within each ventricle population (*n*_LV_ = 10, *n*_RV_ = 14). The error bars correspond to the standard deviation. The average spark amplitude in left ventricle cardiomyocytes was found to be 25% larger than those in right ventricle cardiomyocytes (*p* = 0.0016, UT).

The comparisons for spark rate, amplitude, duration, area and mass as well as transient time-to-half-maximum and dyssynchrony index (defined as the IQR about the median T50 for each cell) for the reduced dataset are shown in Figure 8 and Supplementary Table S8.

Left ventricle cardiomyocyte sparks had a 25% larger amplitude than those in right ventricle cells (0.73 ± 0.12 vs. 0.55 ± 0.12, *p* = 0.0016), while differences in other parameters were not statistically significant. The data exclusion from earlier and later imaging time points resulted in the previously not statistically significant larger spark amplitude for left ventricle being significant, indicating that there may be a gradual change in the cell physiology or environment with time that may affect the spark brightness, resulting in underestimation of the spark amplitude with increasing time since start of loading.

## 4 Discussion

The application of a custom dual-objective light-sheet fluorescence microscope enabled correlative dual-channel 2D time-lapse imaging of calcium dynamics and cellular microstructure in rat ventricular cardiomyocytes. Left and right ventricle cardiomyocytes labelled with a membrane stain and loaded with a calcium indicator were imaged with electrical stimulation and decoupled contraction. Compared to previous approaches, a simpler method involving filtering and thresholding allowed automated and orientation-independent detection of t-tubules. The high spatiotemporal resolution and balance between SNR and photodose enabled detection and characterization of calcium sparks, as well as the determination of transient T50 variation across the cell. By correlating the spark centres with the detected tubule structure, morphological parameters for epitubular and paratubular sparks were compared. Correlating the transient T50 with the tubular structure allowed comparison of transient development dependence on tubule proximity in tubulated and detubulated areas. Finally, spark parameters and transient dyssynchrony in left and right ventricle cardiomyocytes were compared.

### 4.1 Variation in calcium transient development across the cell

The cells were immobilized using para-nitroblebbistatin, a non-phototoxic, lower-fluorescence alternative to the default decoupler blebbistatin, which exhibits phototoxicity and photoinactivation when exposed to blue and UV light (Kolega, 2004). This enabled the implementation of an automated pixel-level analysis of calcium indicator fluorescence time traces during pacing, and hence the mapping of the transient development in 2D without the accompanying motion artefacts from contraction. In line with previous work demonstrating a slower and delayed rise of the calcium transient in the nucleus compared to the cytosol (Ljubojević et al., 2011; Sutanto et al., 2020), the nuclear regions within each cell were found to have a significantly longer T50 than the rest of the cell by 3.47 ms (*p* < 0.0001, WSRT). While nuclear [Ca^2+^] is partially directly regulated by calcium release from its own perinuclear stores, the nuclear transient is dependent on the cytoplasmic calcium concentration due to passive ion diffusion through the nuclear envelope (Ljubojevic and Bers, 2015).

Comparing the T50 in the central part of the cell to the two ends revealed a time-to-half-maximum that was on average 1.97 ms earlier (*p* < 0.0001, WSRT) at the cell ends. The statistical significance was maintained when considering the cell-by-cell differences, averaged for each heart, providing evidence for the dyssynchronous nature of transient development across the cell. Earlier calcium onset has previously been measured at the periphery of atrial myocytes (Hüser et al., 1996; Kockskämper et al., 2001; Mackenzie et al., 2001; Woo et al., 2002), with the less synchronous calcium release in atrial myocytes attributed to the less developed tubular network compared to ventricular myocytes (Berlin, 1995). For ventricular myocytes, this earlier transient onset at the cell ends could be related to the anisotropy of longitudinal cell contraction. Increased displacement and stress at the cell ends during myocyte shortening has previously been measured using traction force microscopy (McCain et al., 2014), and this directionality of contraction may also manifest through earlier calcium release. Hence, additional investigation involving comparison with non-immobilized cells would be of interest.

Correlating the T50 maps with the DNT maps derived from the detected tubules, there was no statistically significant difference between the levels of correlation of transient T50 time with DNT in detubulated and tubulated regions [0.14 ± 0.93 ms/px (mean ± SD) vs. 0.03 ± 0.20 ms/px (mean ± SD)]. Computational modelling by (Marchena and Echebarria, 2020) has suggested reduced synchronization as a result of detubulation in cardiomyocytes, and a positive correlation between T50 and DNT would be expected from previous measurement of transient dyssynchrony in correlation with t-tubule structure (Dries et al., 2013), due to the colocalization of calcium release units with the tubules. However, our study used cardiomyocytes from healthy samples, with a regular t-tubule structure set as one of criteria for cell inclusion in the analysis, and hence in the future, further insight into transient dyssynchrony in detubulated cells could be obtained by looking at cells with more disrupted t-tubule structure, such as those in HF animal models, or induced using formamide (Brette et al., 2002). Additionally, the established imaging and analysis protocols can be extended to compare the calcium dyssynchrony of ventricular myocytes and the lower tubule density atrial myocytes.

### 4.2 Colocalization of calcium sparks and t-tubules

In agreement with previous work (Shacklock et al., 1995; Sikkel et al., 2016), spark centre of mass distribution demonstrated high colocalization with t-tubules, with an average of 63% of sparks in each cell centered on the masked tubules. Besides higher spark rate, using paired statistical tests where parameters are compared for the same cell, epitubular sparks were found to, on average, have significantly longer FDHM (28.0 ± 6.1 ms vs. 25.5 ± 11.1 ms, *p* = 0.016, PT), larger area (6.70 ± 1.91 µm^2^ vs. 5.51 ± 2.34 µm^2^, *p* < 0.0001, WSRT), and larger spark mass (28.1 ± 1.2 ms µm vs. 24.3 ± 13.6 ms µm, *p* = 0.0002, PT) than paratubular sparks, while no significant difference in amplitude between the two spark categories was found.

The co-localization of sparks and tubules is in agreement with previous findings (Shacklock et al., 1995; Cleemann et al., 1998; Sikkel et al., 2016). However, unlike the findings by Sikkel et al. (2016), epitubular sparks were found to have a larger spark area, longer duration and larger spark mass than paratubular sparks. One possible reason for the discrepancy could be due to the minor difference in the spark category definitions: the masked tubules in this work are broader than those generated by Sikkel et al. (2016) and hence more sparks are likely to be categorized as epitubular, skewing the comparison of the differences between the two populations. Additionally, more than two-thirds of the analyzed sparks in the mentioned study were from rats with HF, a condition that may influence the spark morphology of the two categories. Finally, the fact that epitubular sparks, despite having a larger area, duration and signal mass, had no difference in amplitude compared to paratubular sparks, gives confidence that the measured morphology of sparks was minimally skewed by out of focus events, and the larger area for epitubular sparks may be a consequence of epitubular sparks involving joint activation of larger or several RyR clusters colocalized with the t-tubules.

When considering paired cell differences in spark parameters averaged for each animal, only the difference in spark rate remained significant. While this level of testing accounts for potential cardiac heterogeneity, and for the dependence of individual data points on cellular and animal level, the statistical power of this analysis is reduced, and hence it is possible that the other spark parameter differences were not statistically resolved due to the sample size being too small. These challenges can be addressed with higher throughput imaging technology, and more complex multilevel statistical models as described in (Sikkel, 2015) to account for the dependence of measurements on cell and animal level. Additionally, further insight into spark morphology and calcium release dyssynchrony correlation with the underlying cell microstructure can be achieved with localization microscopy of RyR channels, as in the recent implementation by (Hurley et al., 2021).

### 4.3 LV cardiomyocyte sparks have larger amplitude

Comparison of left and right ventricle cardiomyocytes after restricting the dataset to a narrower imaging time window, revealed that left ventricle cells had larger amplitude (0.73 ± 0.12 vs. 0.55 ± 0.12, *p* = 0.0016, UT, *n* = 24). However, the differences in spark rate, duration, area, mass and transient time-to-half-maximum and dyssynchrony were not statistically significant. The reduced spark amplitude in RV myocytes agrees with recent findings by Medvedev made using confocal line-scan measurements (Medvedev, 2020). Other differences between LV and RV identified in Medvedev’s study such as smaller spark mass and higher dyssynchrony index in control RV cells are qualitatively consistent with the results in this work, however, were not found to be statistically significant in our results. This can be due to the smaller sample size and differing methodologies, and further research is needed to verify the results with more certainty. In future work, we recommend that care is taken to ensure that randomisation of experimental groups ensures no bias in their relative imaging time point.

### 4.4 2D LSFM enables quantitative imaging of calcium dynamics with high spatiotemporal resolution

Despite being used widely for studying calcium dynamics in single cell cardiomyocytes, confocal microscopy is disadvantaged by needing to focus the excitation beam to a relatively high intensity spot in the sample. This is unfavourable in terms of phototoxicity and photobleaching and due to the need to record the signal from each pixel sequentially, which requires fast 2D or 3D scanning mechanisms. Line scanning confocal microscopy reduces the excitation intensity experienced by the sample and allows the signal to be recorded from many pixels along the illumination line in parallel, but the illumination signal passes through the whole axial extent of the sample whilst only being detected along one line. In some cases, random access microscopy, where only specific regions of interest are imaged, can allow multiple distinct regions to be sampled rapidly. This method has been applied with two-photon excitation for multisite voltage and calcium recording (Crocini et al., 2014). Light-sheet microscopy has the benefits that the illumination light is used to excite multiple points in the sample along its propagation direction and that a whole 2D image can be acquired in parallel. Previous attempts to characterize the spatial variation in calcium transient development across the cell in 2D using the single-objective-type LSFM method of OPM were limited by cell contraction and low SNR (Maioli, 2016). Compared to this previous work, the dual-objective flexible LSFM system has higher detection NA and detection efficiency, which enables higher spatio-temporal resolution for the same light dose or a lower laser power for equivalent spatio-temporal sampling.

## 5 Conclusion

In this work, a custom dual-objective LSFM was used for high-speed optically sectioned imaging of calcium dynamics in live cardiomyocytes at subcellular resolution. Dual-channel 2D timelapse imaging at 395 fps of electrically stimulated left and right ventricle cardiomyocytes labelled with Fluo-4 and CMO and immobilized with para-nitroblebbistatin enabled the automated quantification and correlation of calcium sparks and transients with the t-tubule structure across *n* = 60 cells from healthy rat hearts. Pixel-wise analysis of the transient time-to-half-maximum demonstrated spatial dyssynchrony in transient development across the cell, with later T50 timepoints in the nucleus and central parts of the cell compared to the rest of the cell and the outer parts respectively. The majority of sparks were found to be colocalized with t-tubules—and those that were not, had, on average, shorter duration, smaller area and spark mass. Left ventricle cardiomyocytes were found to have sparks with larger amplitude.

Overall, the findings indicate that certain spatiotemporal properties of calcium dynamics in cardiomyocytes may spatially correlate with the t-tubule network coverage of the cell, supporting the idea that disruption of the tubular network would introduce changes in spatial dyssynchrony of calcium dynamics across the cell, which could have implications on the contractile strength and uniformity, and hence overall cardiac function. This application of LSFM for imaging millisecond-timescale calcium release events within optical sections of cardiomyocytes demonstrates the technique’s prospect as a fast and high-resolution lower-photodose alternative to 2D confocal microscopy in cardiovascular calcium imaging.

## Data Availability

A representative raw dataset used in this publication is openly available on Zenodo at https://doi.org/10.5281/zenodo.7559264 (Dvinskikh et al., 2023) and the MATLAB-based image analysis script is openly available on Zenodo at https://doi.org/10.5281/zenodo.7561073 (Dvinskikh et al., 2023). The full raw data underlying this publication will be made available upon reasonable request from the corresponding author.
