# Supplementary material for: High-speed 2D light-sheet fluorescence microscopy enables quantification of spatially varying calcium dynamics in ventricular cardiomyocytes
Source: Front Physiol. 2023 Feb 14;14:1079727. doi: 10.3389/fphys.2023.1079727 (PMC9971815; doi:10.3389/fphys.2023.1079727)
Supplement: Supplementary file 1 [file DataSheet1.pdf]

Supplementary Material

1. Methods

1.1 Sample preparation

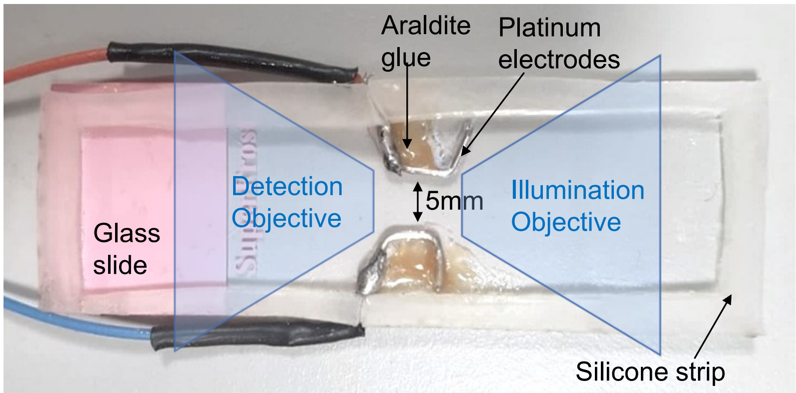

Supplementary Figure 1 Top view of the pacing chamber prototype. The arrangement consists of two platinum 0.8 mm diameter electrodes separated by approximately 5 mm, attached to a glass slide using Araldite glue. A silicone strip around the perimeter of the slide contains the cell media. Approximate objective positioning is illustrated in blue.

1.2 Data pre-processing - cell selection criteria

Cells were considered healthy if they had an elongated brick-like (not curled or rounded) shape, predominantly intact t-tubule structure, no spontaneous contractions or calcium waves during observation, and no change in shape or basal fluorescence during acquisition – the acquisition was excluded if the total variation in baseline fluorescence over the whole acquisition exceeded the transient amplitude. Cells that exhibited mechanical contraction during stimulation were also excluded. Additionally, only cells with transient amplitude of at least 5 digital numbers (DN) above baseline (with an amplitude of approximately $\Delta F/F_0 \geq 1$) were selected, as this was found to correspond to the required SNR for robust automated transient characterization. Finally, each analyzed acquisition was required to have the central part of the cell in focus and no sample drift in any direction.

1.3 Data pre-processing – co-registration, background subtraction and segmentation

Fixed pattern noise estimated from an average of 1000 frames acquired with the laser shutter closed was subtracted from each raw frame in the dataset (representative cell shown in **Supplementary Figure 2**). Next, the two spectral channels were split and co-registered using an affine transformation consisting of rotation, two-dimensional scaling, and translation, with the Fluo-4 images demagnified by ~0.9× in each lateral dimension to account for the magnification introduced by the lenses used to correct for Fluo-4 channel defocus (**Supplementary Figure 2b-c**). The exact parameters for the transformation were manually determined by achieving best visible overlap for a dual-channel dataset

obtained by imaging 200 nm diameter fluorescent beads (T7280, TetraSpeck™, Thermofisher Scientific) embedded in 1% agarose.

For the quantification of calcium dynamics, the Fluo-4 channel background for each individual acquisition was determined by estimating the average signal across a rectangular area manually selected from outside of the cell area. The background was subtracted as a constant offset from each Fluo-4 channel image. Cell segmentation was achieved by intensity thresholding using the Otsu method (Otsu, 1979) of the mean image across 3,160 frames from the Fluo-4 channel, and a series of subsequent modifications of the resultant mask. To exclude any unwanted particles or parts of other cells appearing within the imaged FOV, connected components of the thresholded image containing fewer than 30,000 pixels (corresponding to a cell area of ~650 μm^2) were removed, and the thresholding step was repeated with that region masked out. Any holes within the masked region were filled and the remaining cell mask was smoothed by convolution with a 5×5-pixel x-y kernel. with the resultant cell mask shown as a white outline in the merged channel image in **Supplementary Figure 2d**.

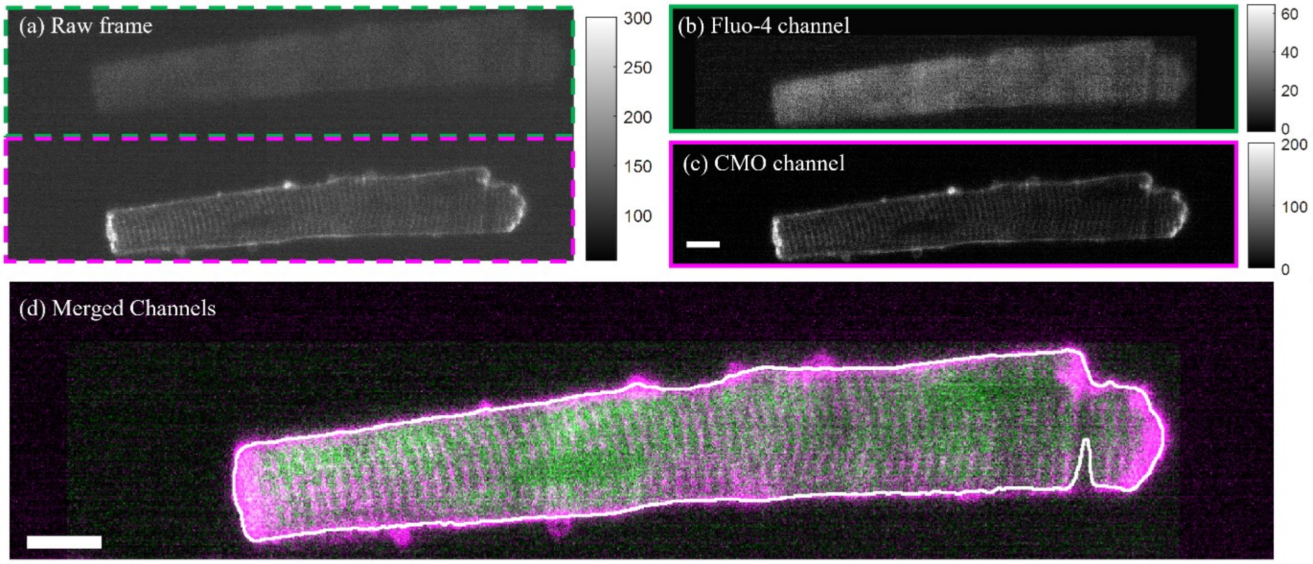

Supplementary Figure 2 Pre-processing of the dual channel data illustrated for a single frame from a 12,000-frame acquisition of representative Cell X at 395 fps at the peak of the first transient. (a) Raw single frame with green and magenta dashed rectangles indicating the ROI used for the Fluo-4 and CMO channels respectively. (b) Separated and co-registered spectral channels with subtracted fixed pattern noise. (c) The Fluo-4 (green) and CMO (magenta) spectral channels overlayed with the segmentation mask outlined in white. Intensities are in digital numbers. Scalebar: 10 μm.

1.4 Image analysis – identifying t-tubule structure and nuclei

As the cell was immobilized and there were minimal motion artefacts during acquisition, an average across 12,000 frames in the CMO-channel was taken to maximize the SNR (**Supplementary Figure 3a**). To account for local background spatially varying across the cell, the average image was blurred by a 15 × 15-pixel median filter, with the resultant image (**Supplementary Figure 3b**) subtracted from the original (**Supplementary Figure 3c**). The tubule microstructure was extracted from the fluorescence of the CMO membrane stain using an intensity threshold. To compensate for the CMO intensity variation between different cells, the threshold used for CMO tubule segmentation was set to 1% of the membrane intensity determined for that cell. The cell membrane intensity was calculated by taking a one-dimensional maximum intensity projection (MIP) along the vertical axis of the CMO image and calculating the average in the horizontal direction across the central third of the FOV (**Supplementary Figure 3d**), which allowed exclusion of the peaks in the vertical MIP at the ends of the cells. The intensity threshold was used to produce a binary mask (**Supplementary Figure 3e**), and connected components with areas less than 20 pixels were excluded, as they were predominantly found to be due to noise artefacts. The tubule mask was multiplied by the cell segmentation mask to exclude any regions outside of the cell and smoothed by a 3 x 3 pixel median filter (**Supplementary Figure 3f).**

Nuclei were segmented by visual inspection of the stained cell membrane in the CMO-channel averaged the across the acquisition. The nuclear envelope was outlined manually using the *drawassisted* MATLAB tool, creating a ROI. The selected ROI were combined to create a binary mask, within which connected components were identified and characterized using the *bwconncomp* and *regionprops* MATLAB tools.

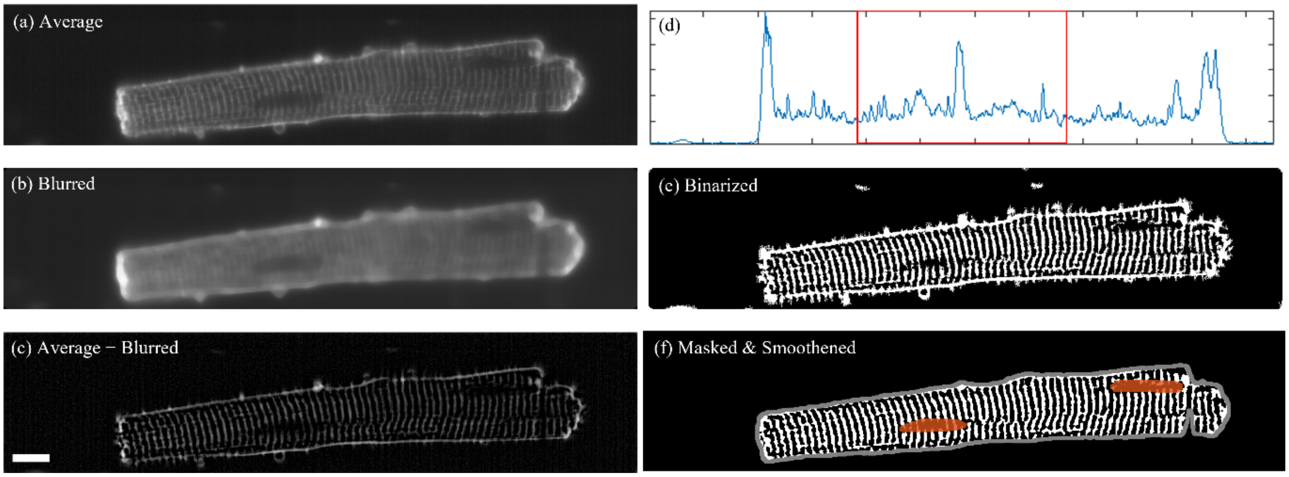

Supplementary Figure 3 Extraction of tubular microstructure from the CMO-channel fluorescence, demonstrated on Cell X. (a) CMO channel image stack average. (b) Average image blurred by a 15 × 15-pixel median filter. (c) Image ‘b’ subtracted from image ‘a’. (d) One-dimensional maximum intensity projection of image ‘a’ taken along the vertical axis, with intensity (a.u.) on the vertical axis and horizontal position (in pixels) on the horizontal axis. The cell membrane intensity is estimated by taking an average within the central third of the horizontal axis (red rectangle outline). (e) Binarized

version of image ‘c’ using an intensity threshold set to 1% of the cell membrane intensity of each cell. (f) Smoothed t-tubule map multiplied by the cell segmentation mask with cell membrane outline shown in grey and the manually identified nuclei in orange. A gamma correction of 0.5 has been applied to the non-binary images a-c to aid visualisation. Scalebar: 10 µm.

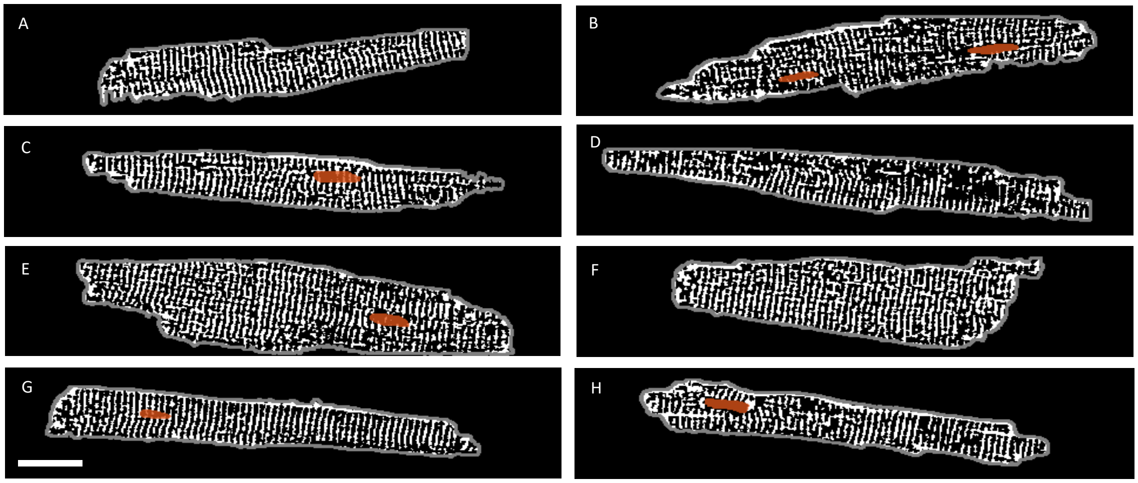

Supplementary Figure 4 T-tubule maps (white) with the cell membrane outline (grey) and manually identified nuclei (orange) across a selection of 8 different cells (A-H). Scalebar: 20 µm.

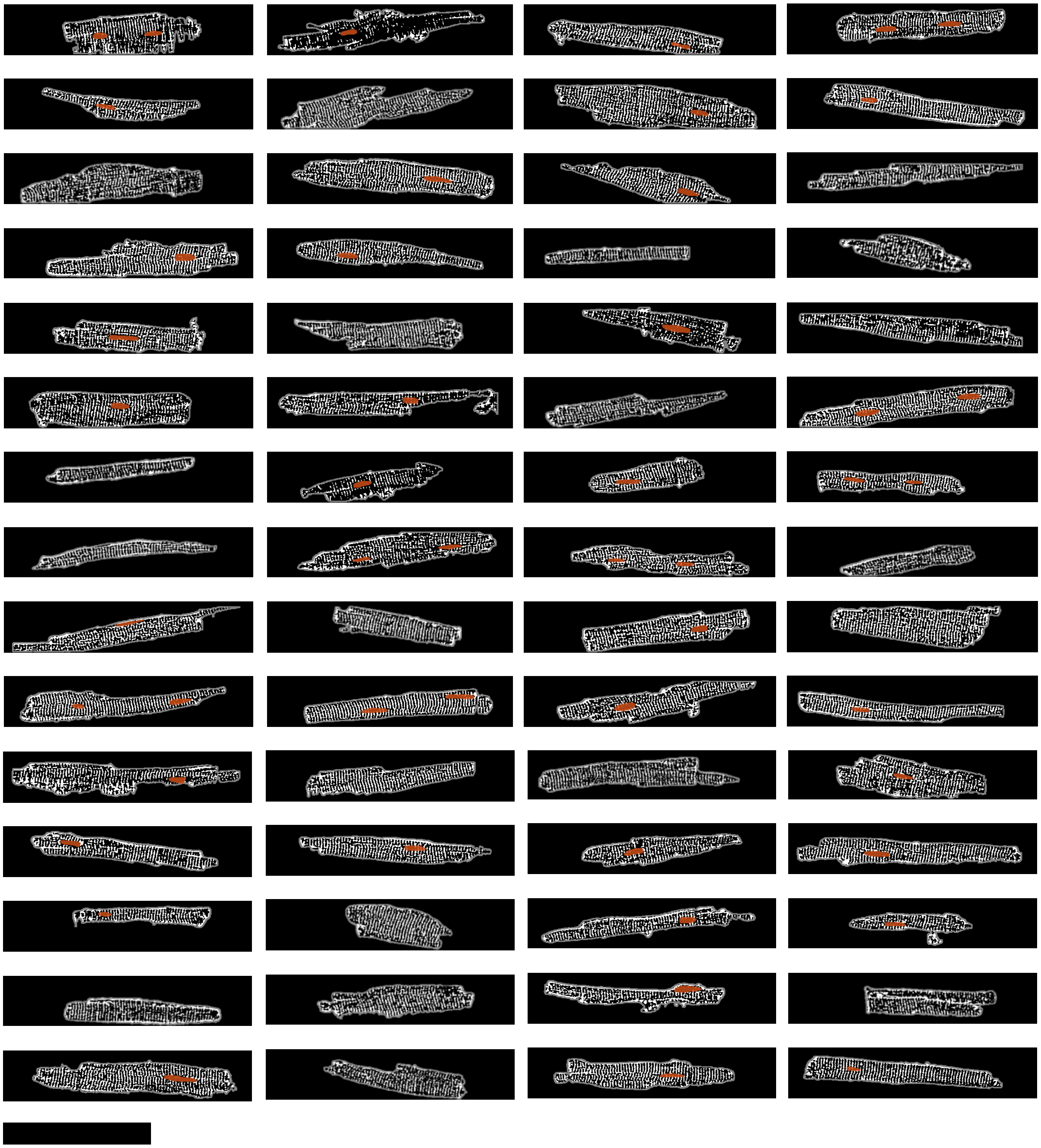

Supplementary Figure 5 T-tubule maps across all analyzed cells with the cell membrane outline shown in grey and manually identified nuclei in orange (n = 60). Scalebar: 100 µm

1.5 Image analysis – transient time-to-half-maximum

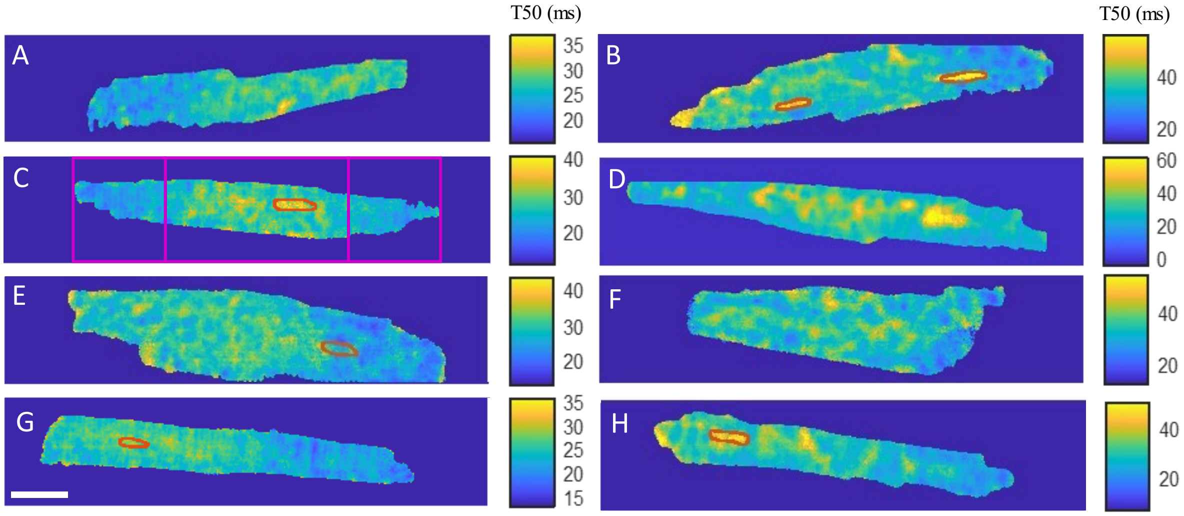

Supplementary Figure 6 Time-to-half-maximum (T50) maps (ms) averaged over three consecutive transients for a selection of cells A-H, with the colormap autoscaled between the median ± (3 × IQR) T50 range, and identified nuclei outlined in orange. The purple rectangles in cell C indicate the centre and edge ROI of the cell. Scalebar: 20 μm

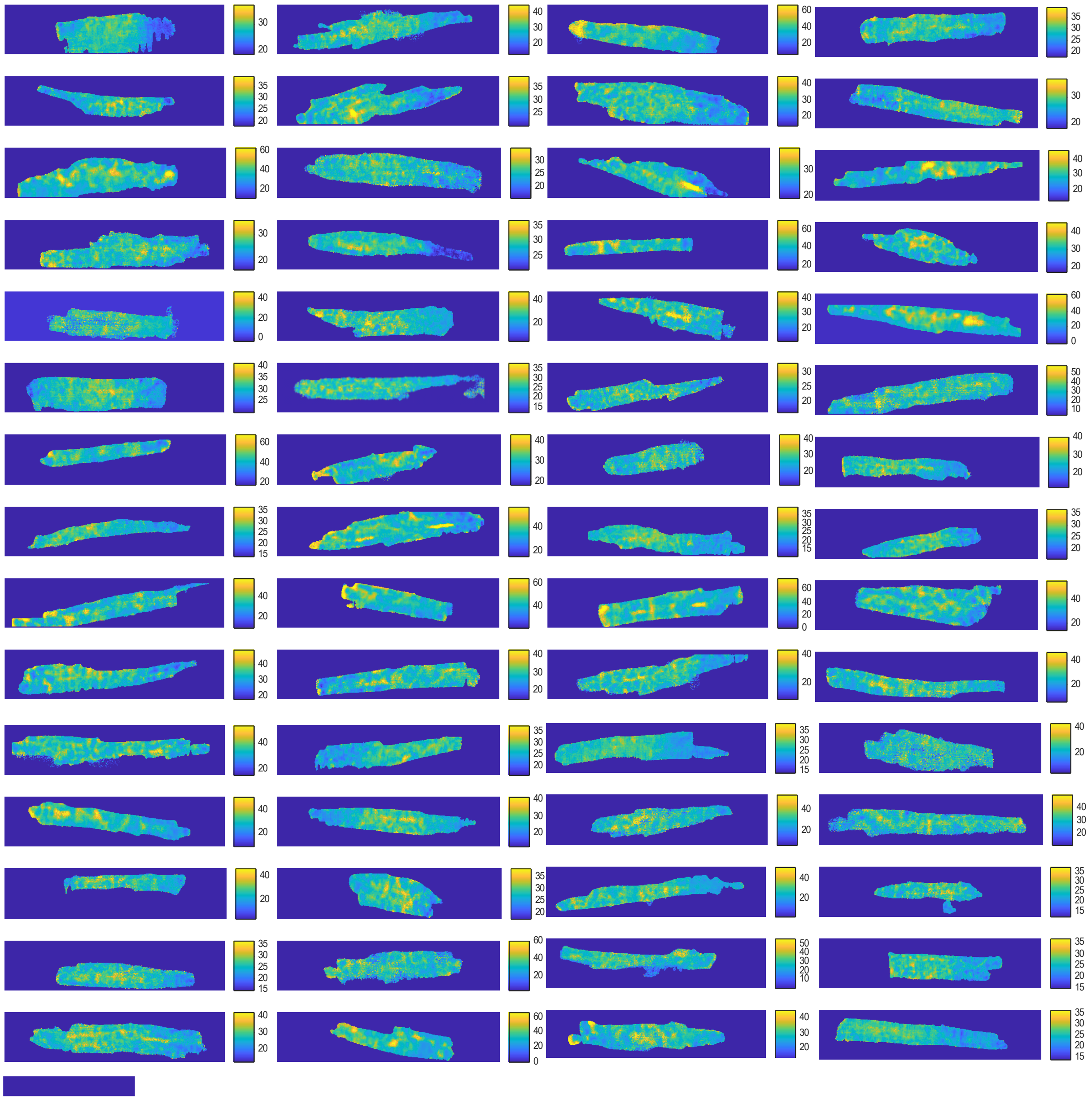

Supplementary Figure 7 Time-to-half-maximum (T50, ms) maps for all $n = 60$ analyzed cells, with the colormap auto-scaled between the median ± IQR values for each cell. Scalebar: 100 µm

2. Results

2.1 Spark characteristics

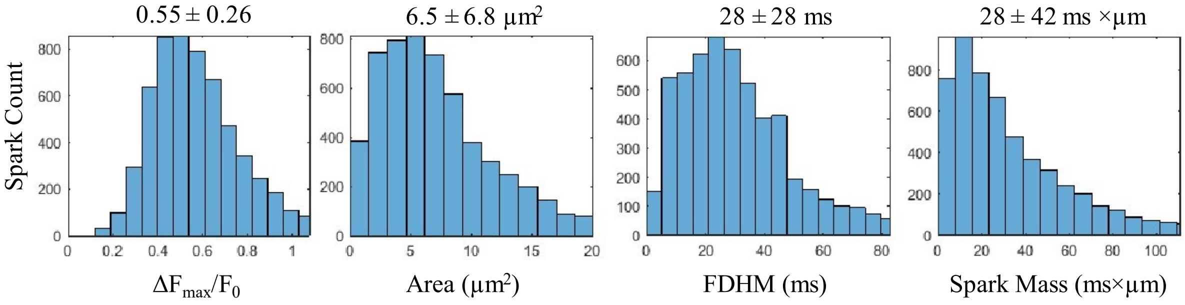

Supplementary Figure 8 Spark count histograms for a selection of calculated parameters (with mean ± SD quoted above) for the total 5,785 sparks detected across $n = 60$ cells. The horizontal axis range is limited up until the median value plus 2×IQR offset.

		DNT = 0	0 < DNT ≤ 5	DNT > 5
**Percentage of total cell area**		38.3%	54.6%	2.9%
Mean	96.42	61.2	33.1	1.58
SD	66.73	44.4	24.17	3.22
SE	8.62	5.7	3.12	0.42

Supplementary Table 1 Total spark number per cell in each distance to nearest tubule (DNT) category, calculated as a mean (with corresponding standard deviation SD and standard error $SE = SD/\sqrt{n}$) across the total number of sparks in each category for each of the $n = 60$ cells. The percentage of total cell area (on average across all analyzed cells) in each DNT category is indicated. The remaining fraction of the cell area (4.2 %) corresponds to nuclear regions.

	Epitubular (DNT = 0)			Paratubular (0 < DNT ≤ 5)			Unpaired		Paired		Heart averages
	Mean	SD	SE	Mean	SD	SE	*p*-value	Test	*p*-value	Test	*p*-value
Spark Rate ($100\mu m^{-2}s^{-1}$)	0.74	0.56	0.07	0.29	0.23	0.03	<0.0001	UT	<0.0001	PT	0.0009
$\Delta F_{max}/F_0$	0.61	0.14	0.02	0.62	0.17	0.02	0.970	UT	0.918	PT	0.425
FDHM (ms)	28.0	6.1	0.8	25.5	11.1	1.4	0.144	UTWC	0.016	PT	0.458
Area (μm^2)	6.70	1.91	0.25	5.51	2.34	0.30	0.006	MW	<0.0001	WSRT	0.149
Spark Mass (ms µm)	28.1	9.2	1.2	24.3	13.6	1.8	0.005	UTWC	0.0002	PT	0.100

Supplementary Table 2 Spark parameter comparison for epitubular (DNT = 0) and paratubular (0 < DNT ≤ 5) sparks with respective standard deviation (SD) and standard errors (SE = SD/√*n*, *n* = 60 cells) calculated from the medians of the spark populations for each cell. Based on considerations outlined in Section 2.4, the *p*-values are calculated using two-sample unpaired *t*-test (UT), unpaired *t*-test with Welch's correction (UTWC), unpaired Mann-Whitney (MW), paired *t*-test (PT), the paired Wilcoxon signed rank test (WSRT), and one-sample *t*-test with cell-by-cell differences averaged by heart. *p*-values <0.05 are highlighted in green, with only the spark rate difference between epitubular and paratubular sparks maintaining statistical significance across all used testing approaches.

2.2 Transient dyssynchrony and correlation with structure

<table>
<tr><th rowspan="2"></th><th colspan="3">T50 (ms)</th><th rowspan="2">Δ (ms)</th><th colspan="2">Unpaired</th><th colspan="2">Paired</th><th>Heart averages</th></tr>
<tr><th>Mean</th><th>SD</th><th>SE</th><th>p-value</th><th>Test</th><th>p-value</th><th>Test</th><th>p-value</th></tr>
<tr><td>Cell</td><td>29.30</td><td>4.63</td><td>0.73</td><td rowspan="2">-3.47</td><td rowspan="2">0.0009</td><td rowspan="2">MW</td><td rowspan="2"><0.0001</td><td rowspan="2">WSRT</td><td rowspan="2">0.00627</td></tr>
<tr><td>Nucleus</td><td>32.77</td><td>7.39</td><td>1.17</td></tr>
<tr><td>Central</td><td>30.07</td><td>4.54</td><td>0.59</td><td rowspan="2">1.97</td><td rowspan="2">0.0018</td><td rowspan="2">MW</td><td rowspan="2"><0.0001</td><td rowspan="2">WSRT</td><td rowspan="2"><0.0001</td></tr>
<tr><td>Outer</td><td>28.10</td><td>4.87</td><td>0.63</td></tr>
</table>

Supplementary Table 3 Comparison of average time-to-half-maximum (T50) for different cellular regions: the cell (excluding the nucleus), the nucleus, the central IQR and the outer quarters. The mean, standard deviation (SD) and standard errors ($SE = SD/\sqrt{n}$) calculated across the medians for each T50 distribution for each of the $n = 40$ cells with identified nuclei for the first category, and all $n = 60$ cells for the second category. The Δ column indicates the difference between the T50 for each region, averaged over all cells. Based on statistical considerations outlined in Section 2.4, the *p*-values are calculated using the unpaired Mann-Whitney (MW), the paired Wilcoxon signed rank test (WSRT), and one-sample nested *t*-test with cell-by-cell differences averaged for each heart. For all three statistical analyses (unpaired, paired by cell, and averaged for each heart) the nuclei and central regions of the cell had a significantly (p-values < 0.05 are highlighted in green) longer time-to half than the rest of the cell and the central region respectively.

<table>
<tr><th rowspan="2"></th><th colspan="3">T50 (ms)</th><th colspan="2" rowspan="2">Δ (ms)</th><th colspan="4">Unpaired</th><th colspan="4">Paired</th><th colspan="2">Heart averages</th></tr>
<tr><td>Mean</td><td>SD</td><td>SE</td><td colspan="2">p-value</td><td colspan="2">Test</td><td colspan="2">p-value</td><td colspan="2">Test</td><td colspan="2">p-value</td></tr>
<tr><td>EpiT</td><td>29.25</td><td>4.76</td><td>0.61</td><td rowspan="2">-0.04</td><td></td><td rowspan="2">0.85</td><td></td><td rowspan="2">MW</td><td></td><td rowspan="2">0.29</td><td></td><td rowspan="2">WSRT</td><td></td><td rowspan="2">0.92</td><td></td></tr>
<tr><td>ParaT</td><td>29.29</td><td>4.54</td><td>0.59</td><td rowspan="2">0.09</td><td rowspan="2">0.92</td><td rowspan="2">MW</td><td rowspan="2">0.18</td><td rowspan="2">WSRT</td><td rowspan="2">0.63</td></tr>
<tr><td>DeT</td><td>29.20</td><td>5.38</td><td>0.69</td><td></td><td></td><td></td><td></td><td></td><td></td></tr>
</table>

Supplementary Table 4 Transient T50 variation with DNT: Comparison of T50 values (ms) within epitubular (DNT = 0), paratubular (0 < DNT ≤ 5) or detubulated (DNT > 5) regions, calculated as an average across the medians for each cell. Positive Δ value indicates that the T50 in the first DNT category is larger than that in the second. The *p*-values are calculated using the unpaired Mann-Whitney (MW), the paired Wilcoxon signed rank test (WSRT), and one-sample nested *t*-test with cell-by-cell differences averaged for each heart. For all three statistical analyses (unpaired, paired by cell, and averaged for each heart) the transient T50 was not significantly different between the DNT categories.

<table>
<tr><th rowspan="2"></th><th colspan="4">ΔT50/ΔDNT (ms/px)</th><th colspan="2">Unpaired</th><th colspan="2">Paired</th><th>Heart Averages</th></tr>
<tr><td>Mean</td><td>95% CI</td><td>SD</td><td>SE</td><td>p-value</td><td>Test</td><td>p-value</td><td>Test</td><td>p-value</td></tr>
<tr><td>ParaT (DNT ≤ 5)</td><td>0.03</td><td>[0.00 0.06]</td><td>0.20</td><td>0.03</td><td rowspan="2">0.75</td><td rowspan="2">MW</td><td rowspan="2">0.5162</td><td rowspan="2">WSRT</td><td rowspan="2">0.32058</td></tr>
<tr><td>DeT (DNT > 5)</td><td>0.14</td><td>[-0.11 0.38]</td><td>0.93</td><td>0.12</td></tr>
</table>

Supplementary Table 5 Comparison of correlation slopes (ΔT50/ΔDNT, including 95% confidence intervals (CI) of the linear fit) in tubulated (DNT < 5) and detubulated (DNT > 5) cell areas, calculated as an average across the slopes for each cell. The *p*-values are calculated using the unpaired Mann-Whitney (MW), the paired Wilcoxon signed rank test (WSRT), and one-sample nested *t*-test with cell-by-cell differences nested by heart. For all three statistical analyses (unpaired, paired by cell, and nested by heart) the T50/DNT slope was not significantly different between the two DNT categories.

2.3 Comparison of left and right ventricle cardiomyocytes

	**LV (*n* = 32 Cells)**			**RV (*n* = 28 Cells)**			**Δ (%)**	***p*-value**	***t*-test**
	Mean	SD	SE	Mean	SD	SE			
Spark Rate (100 $\mu m^{-2} s^{-1}$)	0.48	0.33	0.04	0.41	0.24	0.05	14	0.94	UT
Spark $\Delta F_{max}/F_0$	0.64	0.15	0.03	0.59	0.14	0.03	7	0.23	UT
Spark FDHM (ms)	26.8	8.0	1.4	27.5	6.4	1.2	-3	0.75	UT
Spark Area (μm^2)	6.12	1.68	0.28	6.39	2.15	0.41	-4	0.76	UT
Spark Mass (ms×µm)	27.0	10.1	1.8	26.3	9.0	1.7	3	0.79	UT
Transient T50 (ms)	28.4	4.2	0.7	30.3	4.9	0.9	-6.7	0.09	MW
Transient DI (ms)	5.42	2.29	0.40	6.19	2.37	0.45	-14	0.21	MW

Supplementary Table 6 Comparison of the spark and transient parameters calculated for LV and RV CM the full $n = 60$ dataset quoted as the mean, standard deviation (SD) and standard errors ($SE = SD/\sqrt{n}$) calculated from the medians of the cell parameters in each of the two cell populations. The differences (Δ) are calculated as the difference between corresponding parameters in left and right ventricles, divided by the former value, and converted to a percentage. The *p*-values are calculated using the unpaired *t*-test (UT) and the unpaired Mann-Whitney (MW). For the full $n = 60$ dataset there were no significant differences across any of the spark parameters.

To evaluate the potential systematic bias in imaging time due to un-randomized blinding of the two ventricles, the correlation of each parameter with the relative imaging time point (to the first imaged cell in that batch) was considered. Correlation was assessed by linear fits to the data for cells from each ventricle and considering the coefficient of determination R^2. The slopes (Δ), vertical axis intercepts (Y_0) and R^2 coefficients for the linear fits are summarized in **Supplementary Table 7.** A weak correlation with the time of imaging was found for some spark and transient parameters ($R^2 = 0.12$-0.49), with a positive time correlation for spark rate and FDHM in both ventricles and LV respectively, and a negative time correlation for spark amplitude and transient dyssynchrony for LV CM and RV CM respectively.

The scatterplots for parameters with a weak correlation ($R^2 > 0.1$) present are shown in **Supplementary Figure 7.** Two of the selected left ventricle acquisitions had a significantly earlier relative imaging time point compared to the rest (0 and 7 min respectively). To evaluate the influence of these outliers on the time correlation of spark amplitude, the linear correlation was calculated for the dataset excluding those two points (**Supplementary Figure 8**). As a result, the slope and intercept of the new linear correlation fit remained nearly unchanged ($\Delta = -0.003$, $Y_0 = 0.93$), while the coefficient of determination decreased from $R^2 = 0.49$ to $R^2 = 0.31$, indicating a lower negative correlation level, but with a similar time dependence.

	LV (n = 32 Cells)			RV (n = 28 Cells)			Combined		
	Δ	Y_0	R^2	Δ	Y_0	R^2	Δ	Y_0	R^2
Spark Rate (100 $\mu m^{-2} s^{-1}$)	0.005	0.05	0.15	0.004	0.27	0.13	0.003	0.24	0.12
Spark $\Delta F_{max}/F_0$	-0.003	0.94	0.49	5×10^{-5}	0.59	<0.001	-0.001	0.67	0.05
Spark FDHM (ms)	0.090	18.7	0.13	0.03	26.2	0.014	0.03	24.9	0.03
Spark Areas (μm^2)	0.001	5.16	0.046	-0.003	6.49	<0.001	0.001	6.17	<0.001
Spark Mass (ms×µm)	0.013	25.8	0.002	0.010	26.0	0.001	0.013	25.9	0.003
Transient T50 (ms)	-0.018	30.0	0.02	-0.036	31.7	0.033	-0.03	31.3	0.06
Transient DI (ms)	-0.002	5.60	<0.001	0.034	4.83	0.12	-0.001	5.83	<0.001

Supplementary Table 7 Correlation of the calculated spark and transient parameters with relative imaging time point. The slope (Δ), y-axis intercept (Y_0) and the coefficient of determination (R^2) are given above, with R^2 >0.1 highlighted in yellow and R^2 > 0.3 in green. The spark rate of LV, RV and combined cardiomyocytes may have weak positive correlation with imaging time. The transient dyssynchrony index of RV cardiomyocytes also exhibits weak positive correlation with imaging time. Spark amplitude of left ventricle CM had stronger positive correlation with imaging time (R^2 = 0.49).

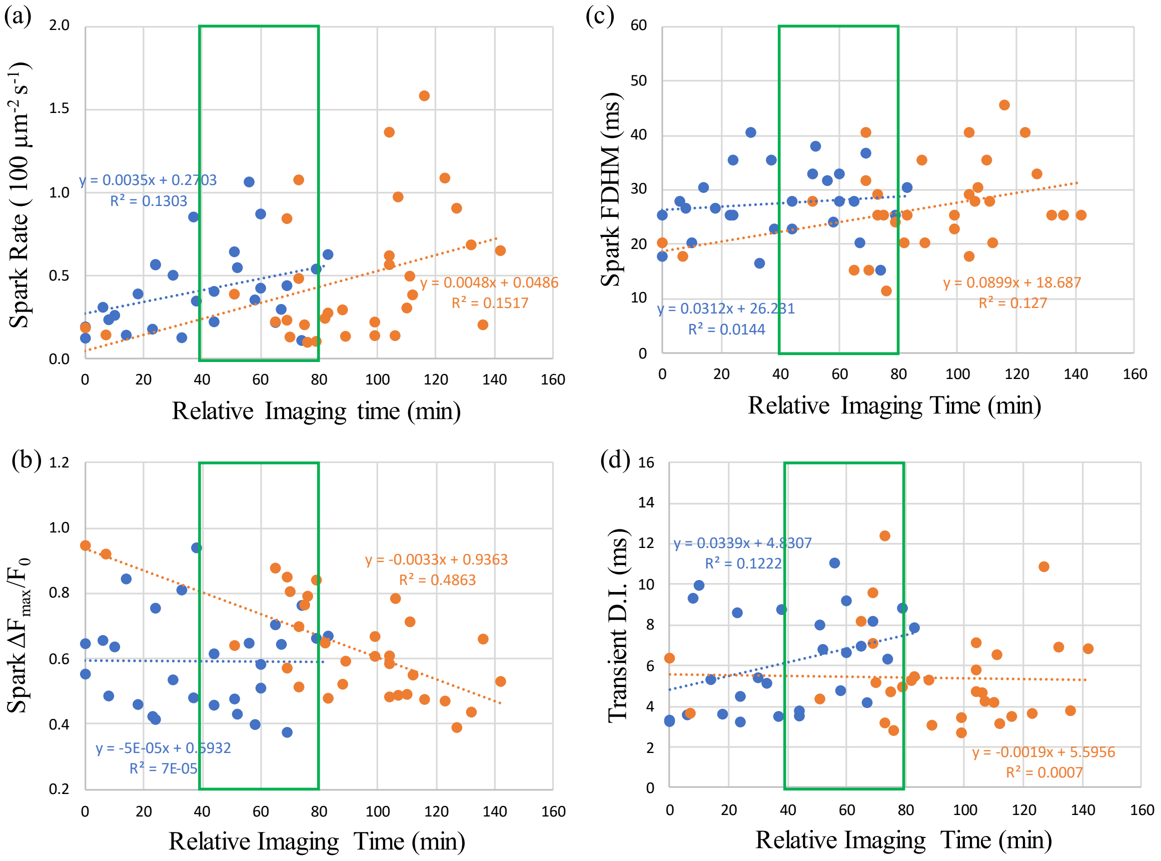

Supplementary Figure 9 Correlation of selected spark and transient parameter medians for each cell ($n = 60$) with the relative time of imaging for left (orange) and right (blue) ventricle cardiomyocytes. The equation and R-squared (R^2) coefficients of determination for the linear fits are provided next to the datapoints. The green rectangles indicate the time window selected for the reduced dataset ($n = 24$, $n_{LV} = 10$, $n_{RV} = 14$ cells).

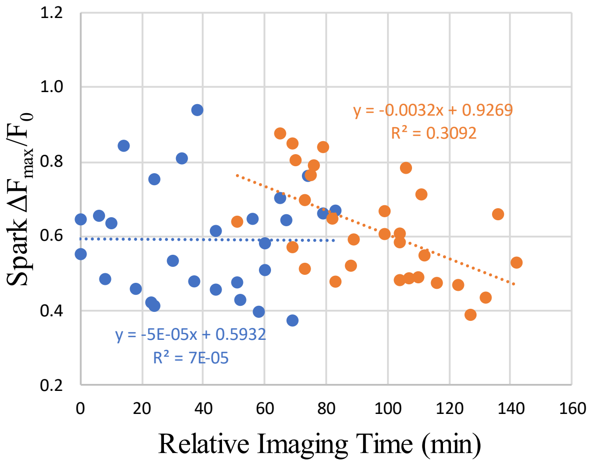

Supplementary Figure 10 Correlation of spark amplitude with the relative time of imaging for LV (orange) and RV (blue) CM, with two earliest LV CM datapoints excluded. The equation and R-squared (R^2) coefficients of determination for the linear fits are provided next to the datapoints.

	**All (*n* = 24 cells)**			**LV (*n* = 10 cells)**			**RV (*n* = 14 cells)**			**Δ (%)**	***p*-value**
	Mean	SD	SE	Mean	SD	SE	Mean	SD	SE		
Spark Rate (100μm^{-2} s^{-1})	0.44	0.28	0.06	0.38	0.32	0.10	0.48	0.25	0.07	-27	0.415
Spark $\Delta F_{max}/F_0$	0.63	0.15	0.03	0.73	0.12	0.04	0.55	0.12	0.03	25	0.0016
Spark FDHM (ms)	26.9	7.5	1.5	24.6	8.3	2.6	28.5	6.3	1.7	-16	0.22
Spark Area (μm^2)	5.98	1.56	0.32	5.62	1.29	0.41	6.23	1.68	0.45	-11	0.36
Spark Mass (ms×μm)	26.6	9.9	2.0	28.3	11.8	3.7	25.4	8.1	2.2	10	0.50
Transient T50 (ms)	29.3	4.9	1.0	29.8	6.0	1.9	29.0	3.7	1.0	2.7	0.70
Transient DI (ms)	6.46	2.51	0.51	6.24	2.88	0.91	6.62	2.19	0.59	-6.1	0.73

Supplementary Table 8 Comparison of the spark parameters and transient T50 calculated for left and right ventricle cardiomyocyte populations from a sub-dataset within a restricted time window (n = 24 cells), quoted as the mean, standard deviation (SD) and standard errors (SE = SD/$\sqrt{n}$) calculated from the medians of the cells in each of the two populations. The differences (Δ) are calculated as the average for each parameter for LV myocytes minus the average for RV myocytes, divided by the former value, and converted to a percentage. Positive Δ indicates larger value for LV myocytes. All datasets were normally distributed, and each dataset pair passed the F-test for equal variance, and hence p-values were calculated using two sided, two sample, homoscedastic unpaired t-test (UT). For the calculated parameters, only the spark amplitude was significantly different between the two ventricles, with LV CM having 25% larger spark amplitude (p = 0.0016).

Supplementary Video 1 2D LSFM timelapse of a single ventricular cardiomyocyte undergoing stimulated calcium transients and spontaneous sparks, imaged at 395 fps. The cell is electrically paced for around half of the acquisition. Video has been down sampled by 16x in time through bilinear interpolation. Rendered with JPEG compression. Playback at 2× real-time speed. Scalebar: 10 µm.

References:

Otsu, N. (1979) A Threshold Selection Method from Gray-Level Histograms. *IEEE Trans. Syst. Man Cybern. Syst* 9 (1), 62-66. doi: 10.1109/TSMC.1979.4310076.
